# Genomic Characteristics and Comparative Genomics Analysis of Two Chinese *Corynespora cassiicola* Strains Causing Corynespora Leaf Fall (CLF) Disease

**DOI:** 10.3390/jof7060485

**Published:** 2021-06-16

**Authors:** Boxun Li, Yang Yang, Jimiao Cai, Xianbao Liu, Tao Shi, Chaoping Li, Yipeng Chen, Pan Xu, Guixiu Huang

**Affiliations:** 1 Environment and Plant Protection Institute, Chinese Academy of Tropical Agricultural Sciences, 4 Xueyuan Road, Haikou 571101, China; diyningxiang@126.com (B.L.); yangyang@catas.cn (Y.Y.); cjmiao1231@163.com (J.C.); liuxianbao2013@126.com (X.L.); shitaofly2008@163.com (T.S.); lichaoping2008@163.com (C.L.); cypkiller@163.com (Y.C.); 2 College of Grassland Agriculture Science and Technology, Lanzhou University, 222 Tianshui South Road, Lanzhou 730000, China; 3 Key Laboratory of Integrated Pest Management on Tropical Grops, Ministry of Agriculture and Rural Affairs, Beijing 100020, China; digers_xup900104@163.com

**Keywords:** comparative genomics, *Corynespora cassiicola*, pathogenicity, Corynespora leaf fall disease, rubber tree, secondary metabolites

## Abstract

Rubber tree *Corynespora* leaf fall (CLF) disease, caused by the fungus *Corynespora cassiicola*, is one of the most damaging diseases in rubber tree plantations in Asia and Africa, and this disease also threatens rubber nurseries and young rubber plantations in China. *C. cassiicola* isolates display high genetic diversity, and virulence profiles vary significantly depending on cultivar. Although one phytotoxin (cassicolin) has been identified, it cannot fully explain the diversity in pathogenicity between *C. cassiicola* species, and some virulent *C. cassiicola* strains do not contain the cassiicolin gene. In the present study, we report high-quality gapless genome sequences, obtained using short-read sequencing and single-molecule long-read sequencing, of two Chinese *C. cassiicola* virulent strains. Comparative genomics of gene families in these two stains and a virulent CPP strain from the Philippines showed that all three strains experienced different selective pressures, and metabolism-related gene families vary between the strains. Secreted protein analysis indicated that the quantities of secreted cell wall-degrading enzymes were correlated with pathogenesis, and the most aggressive CCP strain (cassiicolin toxin type 1) encoded 27.34% and 39.74% more secreted carbohydrate-active enzymes (CAZymes) than Chinese strains YN49 and CC01, respectively, both of which can only infect rubber tree saplings. The results of antiSMASH analysis showed that all three strains encode ~60 secondary metabolite biosynthesis gene clusters (SM BGCs). Phylogenomic and domain structure analyses of core synthesis genes, together with synteny analysis of polyketide synthase (PKS) and non-ribosomal peptide synthetase (NRPS) gene clusters, revealed diversity in the distribution of SM BGCs between strains, as well as SM polymorphisms, which may play an important role in pathogenic progress. The results expand our understanding of the *C. cassiicola* genome. Further comparative genomic analysis indicates that secreted CAZymes and SMs may influence pathogenicity in rubber tree plantations. The findings facilitate future exploration of the molecular pathogenic mechanism of *C. cassiicola*.

## 1. Introduction

The fungus *Corynespora cassiicola* (Berk. and Curt) C. T. Wei, belonging to the Ascomycota phylum, Dothideomycetes class, and Pleosporales order, is responsible for diseases in a wide range of plants, including rubber tree, tomato, cucumber, soybean, cotton, and various others [1]. This fungus has also been isolated from sponges, nematodes, and rare human infections [2,3,4]. *C. cassiicola* was once considered a weak pathogen of rubber trees (*Hevea brasiliensis*, Muell. Arg.), until Corynespora leaf fall (CLF) disease was first reported in Sierra Leone, which devastated more than 4000 ha of the highly susceptible rubber cultivar RRIC103 in the 1980s [5,6]. Since then, CFL disease has rapidly spread through most rubber-producing countries in Asia and Africa, causing severe sporadic epidemics and damaging losses to natural rubber yields, making it among the most important cryptogamic diseases of rubber plantations [7].

CLF disease is characterised by the development of necrotic lesions on leaves, with frequent blackening of the veins, resulting in a typical ‘fish-bone’ or ‘railway track’ appearance. Both immature and mature leaves are affected, leading to massive defoliation and consequent growth delay and yield losses [8]. In China, CLF disease was first detected in 2006 in rubber nurseries and young rubber plantations in the Yunnan and Hainan provinces [9]. The rubber tree is native to the Amazon basin, but historically it has been widely cropped in tropical regions within equatorial zones (between 10° N and 10° S) [10]. With increasing demand for natural rubber, rubber plantations expanded rapidly through the 20th century in China [11], and China is now the world’s third largest rubber plantation region, with a planting area of 1.18 million ha (ANRPC). Unlike other rubber-producing countries, most rubber plantations in China are scattered between 18° N and 24° N, and some rubber plantations in the Yunnan province have expanded into marginal growing environments as far north as 24° N and at high elevations exceeding 1300 m [12]. Differences in geographical environment and climate variation can lead to genetic variation between fungal strains. *C. cassiicola* strains sequenced in this study were separately isolated from rubber plantations in Hekou, the Yunnan province (strain YN49), and Yangjiang, the Guangdong province (strain CC01). The straight-line distance between the two places is more than 800 kilometres, and they have very different ecological environment and weather conditions. 

Cassiicolin, a small 27 amino acid secreted glycoprotein with six cysteines engaged in three disulphide bonds, is the only characterised *C. cassiicola* effector to date [13]. Six different cassiicolin isoforms (Cas1–6) have been described from different *C. cassiicola* isolates sampled from various hosts and geographical origins, and some lack the cassiicolin-encoding gene (Cas0 isolates) [14]. According to one study [14], Cas1 is the major rubber tree-specialised isolate in the Philippines, while Cas3, 4 and 5 are the most abundant in Malaysia, and Cas5, Cas0 and Cas2 predominate in China, and Cas2 types are only found in cucumber and soybean in other countries [15]. These results indicate that *C. cassiicola* strains evolved independently due to geographical factors. 

Interestingly, Cas0 isolates, which have no cassiicolin-encoding gene, can also cause CFL in rubber trees and other plants, such as tomato, pepper and papaya [14,16]. This indicates that a variety of pathogenic factors remain to be identified in *C. cassiicola*. The cell wall, which comprises a matrix of pectin, hemicellulose, lignin and structural proteins, is an important barrier that protects plants from pathogen attack [17]. In rubber trees, a thick cuticle and wax layer improves resistance to phytopathogenic fungi [18]. To overcome this barrier, phytopathogenic fungi produce cell wall-degrading enzymes (CWDEs) that degrade cellulose, xylan, pectin and cell wall polymers [19]. Lopez et al. (2018) analysed proteins secreted from *C. cassiicola* and found that this fungus secretes a variety kinds of degradatory enzymes, including proteases, carbohydrate-active enzymes (CAZymes) and lipases [16]. Meanwhile, Liu et al. (2011) found that *C. cassiicola* secretes CWDEs as pathogenic factors, including cutinase, as well as cytomembrane- and cell inclusion-degrading enzymes [20]. However, whether and how secreted proteins affect the pathogenicity of *C. cassiicola* toward rubber trees remains unclear.

*C. cassiicola* CCP, a highly virulent strain collected from diseased rubber trees in the Philippines, is the only *C. cassiicola* strain for which the whole genome has been sequenced, assembled and annotated [16,21]. Lopez et al. (2018) identified 63 secondary metabolite biosynthesis gene clusters (SM BGCs) in CCP, far more than in other phytopathogenic fungi (~40) [16]. Filamentous fungi produce a diverse array of SMs that function as toxins, antibiotics and pigments [22]. In phytopathogenic fungi, a variety of SMs are produced during interactions with plants, and used as weapons to invade host plants [23]. SM biosynthetic genes in filamentous fungi are typically organised into contiguous gene clusters in the genome, and clustered near chemical backbone synthesis genes, such as non-ribosomal peptide synthases (NRPSs), polyketide synthases (PKSs) and terpene synthases [24]. In contrast to obligate biotrophic fungi, necrotrophic and hemibiotrophic fungi often contain many SM BGCs [23]. However, synthetic products of SM gene clusters in *C. cassiicola* and their roles in pathogenesis remain unclear. SM gene clusters vary between *C. cassiicola* strains, and their relevance to pathogenicity requires further study. 

In the present study, we determined gapless genome sequences of two highly virulent *C. cassiicola* strains that cause CFL disease in rubber trees in China. This is the first genome reported for a Chinese CFL disease-causing strain. In order to analyse pathogenesis-related genes and pathways, we compared Chinese strains and a virulent reference isolate CCP genome. The results showed that all three strains evolved under different selective pressures, and possess different secreted CAZymes and SM gene cluster compositions. This indicates that secreted CAZymes and SMs may influence pathogenicity in fungi affecting rubber trees, and may also partially explain the high variability in the pathogenicity of *C. cassiicola*.

## 2. Materials and Methods

### 2.1. Fungal Growth Conditions and DNA Preparation

*C. cassiicola* YN49 (CGMCC 3.20259) and CC01 (CGMCC 3.20258) strains were separately isolated from rubber plantations in Hekou, Yunnan province, and Yangjiang, Guangdong province. Both strains were maintained in our laboratory. Fungi were grown on potato dextrose agar medium and incubated at 28 °C for 10 days. Mycelia were harvested and DNA was extracted from grounded mycelia using a genomic DNA kit (Qiagen, New York, NY, USA). Agarose gel electrophoresis, a NanoDrop 1000 spectrophotometer (Thermo, Bedford, MA, USA) and a Qubit fluorimeter (Thermo, Bedford, MA, USA) were used to analyse the integrity, quality, and concentration of DNA, respectively. Genomic DNA was further purified for sequencing (Oxford Nanopore Technology, Oxford, UK) using a BluePippin DNA size selection system (Sage Science, Beverly, MA, USA). 

### 2.2. Pathogenicity Tests

Rubber tree cultivar Reyan7-33-97, one of the main rubber varieties in China, was selected to analysis pathogenicity of *C. cassiicola* YN49 and CC01 according to protocol of Déon’s with modifications [14]. The *C. cassiicola* isolates were cultivated on PDA medium at 28 °C and conidia were collected and resuspended in sterile water at a concentration of 10,000 conidia mL^−1^. Three drops of each conidial suspension (20 μL) were applied to the abaxial surface of 1 detached rubber tree leaflet at developmental stage C (brownish to limp green) and leaflets were maintained in a moist environment at 28 °C for 24 h in the dark and then under alternate light (photoperiod 12 h/12 h).

### 2.3. Detection and Sequencing of the Cassiicolin-Encoding Genes

Detection of the cassiicolin gene was conducted by PCR on genomic DNA from *C. cassiicola* isolates YN49 and CC01. Primers and PCR reaction conditions of Déon’s were used for cassiicolin gene detection [14]. PCR product of cassiicolin genes was sequenced by BGI (Shenzhen, China). Maximum likelihood trees generated using amino acid sequences of A and KS domains were constructed using the method described previously by Déon et al. (2014) with MEGA version 6.0.

### 2.4. Genome Sequencing and Assembly

After repairing DNA damage, an SQK-LSK108 ligation kit (Oxford Nanopore Technology, UK) was used to construct a library, and a Qubit fluorimeter was used to assess the quality of the library. Single-molecule real-time sequencing of long reads was conducted on a GridION X5 platform (Oxford Nanopore Technology, UK). After filtering the sequencing adapters and low-quality sequences, clean data (YN49, 5.1 Gb clean reads, 115 × sequencing depth; CC01, 10.5 Gb clean reads, 228× sequencing depth) were obtained and assembled using CANU (version 1.3) [25] with default parameters. In addition, a separate paired-end (PE) DNA library was sequenced using an Illumina HiSeq4000 platform (Illumina, San Diego, CA, USA). The sequencing data (filtered reads = 2.99 Gb, sequencing depth = 90×) were used to further improve the assembly using bwa mem (version 0.7.12-r1039) and two runs of Pilon (version 1.22) with continuous iteration correction [26]. The integrity of assembly was evaluated using BUSCO (benchmarking universal single-copy orthologs) version 3.0.1 (https://busco.ezlab.org/ accessed on 13 October 2020) [27].

### 2.5. Gene Prediction and Annotation

A combination of Augustus and Glimmer was used for de novo prediction of protein-coding genes by constructing models. GeneWise [28] was then used to predict protein-coding genes via homology analysis with known protein sequences from related species (*C. cassiicola*, *Cercospora canescens*, *Bipolaris maydis*, *Pyricularia oryzae*, *Fusarium oxysporum* and *Pseudocercospora fijiensis*). EVidenceModeler (EVM) (http://evidencemodeler.github.io/ accessed on 13 October 2020) was subsequently used to compute weighted consensus gene structure annotations. We obtained the final gene sets after removing genes with transposable elements using TransposonPSI (http://transposonpsi.sourceforge.net/ accessed on 13 October 2020). Functional annotations for all predicted gene models were made using multiple databases, including Swiss-Prot, NR, KEGG and COG, and BlastP, with E-values ≤ 1.0 × 10^−5^.

### 2.6. Orthologous Gene Family, Phylogenetic, and Whole-Genome Synteny Analyses

OrthoMCL [29] was used for analysis of orthologous gene families (E-values ≤ 1.0 × 10^−5^) by comparing proteins from *C. cassiicola* YN49 and CC01 with those of the following 10 phytopathogenic fungi: *C. cassiicola* CCP (GCA_003016335.1), *B. maydis* (GCA_000338975.1), *C. zeae-maydis* (2761,201,826 JGI), *Parastagonospora nodorum* (GCA_002267025.1), *Exserohilum turcica* (GCA_000359705.1), *Pyrenophora tritici-repentis* (GCA_000149985.1), *P. oryzae* (GCA_000002495.2), *Aspergillus flavus* (GCA_009017415.1), *F. graminearum* (GCA_000240135.3) and *Botrytis cinerea* (GCA_000143535.4). Phylogenetic relationships between *C. cassiicola* YN49, CC01 with the other 10 phytopathogenic fungi were analysed using single-copy orthologous gene groups. Gblocks (with default parameters) were used to remove divergence and ambiguously aligned blocks from the alignment to obtain a better CDS file. A maximum likelihood tree was constructed using RaxML [30] with the GTRGAMMA model and 100 bootstrap replicates to infer phylogenetic relationships. Synteny blocks between *C. cassiicola* YN49, CC01 and CCP were identified and represented by minimap2 (version 2.17) with the cx asm5 parameter [31].

### 2.7. Gene Family Expansion Analysis

Using the OrthoMCL gene family results, CAFE (computational analysis of gene family evolution, version 4.0.1) [32] was employed to detect gene family expansion and contraction (using divergence time instead of branch length).

### 2.8. Positive Selection

To perform positive selection, we obtained a new gene set of orthologous gene pairs using the genomes of the 12 phytopathogenic fungi mentioned before. Using BLAST (version 2.2.30) with an E-value cut-off ≤ 1.0 × 10^−5^, we identified orthologous gene pairs with reciprocal best hits among the 12 species. We estimated the dN/dS ratio (ω) using PAML version 4.9e [33] with the coding sequence alignments above to determine the selection pressure on corresponding gene pairs. Genes with positively selected sites were detected using branch-site models (model  =  2 and NSsite  =  2). For the null hypothesis we used the parameters fix_omega = 1 and omega  =  1, but for the alternative hypothesis we used fix_omega  =  0 and omega  =  1.5. We used a false discovery rate (FDR)-corrected likelihood ratio test (LRT) with an adjusted LRT *p*-value cut-off ≤ 0.05 to identify positively selected sites of genes.

### 2.9. Annotation of Specific Gene Categories

The following five different methods were used to identify repetitive sequences: RepeatProteinMasker (version 1.36), RepeatMasker (version 4.0.7) [34], TRF, RepeatModeler (http://repeatmasker.org/RepeatModeler/ accessed on 13 October 2020), and Ltr-finder [35]. CAZymes were predicted in YN49, CC01 and CCp strain genomes by performing a BLASTP search (E-values ≤ 1.0 × 10^−5^) against the Carbohydrate-Active EnZYmes (CAZy) database (http://www.cazy.org/ accessed on 13 October 2020) with predicted protein sequences as queries [36].

Lipases, proteases and the secretome were predicted according to the procedure described previously by Lopez and colleagues for *Corynespora cassiicola* [16]. Hmmsearch (HMMER 3.1b1; http://hmmer.org/ accessed on 13 October 2020) was used with predicted protein sequences as queries to search against the Lipase Engineering Database (version 3) with an E-value inclusion threshold set at 0.01 [37]. Proteases were predicted in YN49, CC01 and CCp strain genomes by performing a BLASTP search (E-values ≤ 1.0 × 10^−5^) against the MEROPS blast database (version 9.12) with predicted protein sequences as queries [38]. SignalP (http://www.cbs.dtu.dk/services/SignalP/ accessed on 13 October 2020) was used to predict signal peptides and cleavage sites of predicted proteins. Proteins with a signal P D-score = Y were scanned for transmembrane spanning regions using TMHMM (version 2.0; http://www.cbs.dtu.dk/services/TMHMM/ accessed on 13 October 2020) and all proteins with 0 TMs or 1 TM, if located in the predicted N-terminal signal peptide, were retained. Proteins potentially secreted through endoplasmic reticulum (ER)/Golgi-independent pathways were not taken into account in this study.

### 2.10. Secondary Metabolite Gene Cluster Analysis

Genes encoding putative polyketide synthases (PKSs), non-ribosomal peptide synthases (NRPSs), PKS–NRPS hybrids, terpene synthases (TSs), and their modules were identified by searching the antiSMASH database (fungal version; https://fungismash.secondarymetabolites.org/ accessed on 13 October 2020) with default settings [39]. Genes putatively involved in secondary metabolism were also identified with antiSMASH. Maximum likelihood trees generated using amino acid sequences of A and KS, and domains were constructed using the method described previously by Yang et al. (2019) with MEGA version 6.0 employing the Wheland and Goldman (WAG) mode [40]. Then, iTOL (v5) was used to annotate phylogenetic trees (https://itol.embl.de/index.shtml accessed on 13 October 2020).

## 3. Results

### 3.1. C. cassiicola Cas5 and Cas2 Isolates from China Differ in Virulence toward Rubber Tree Leaves

**Figure 1:**
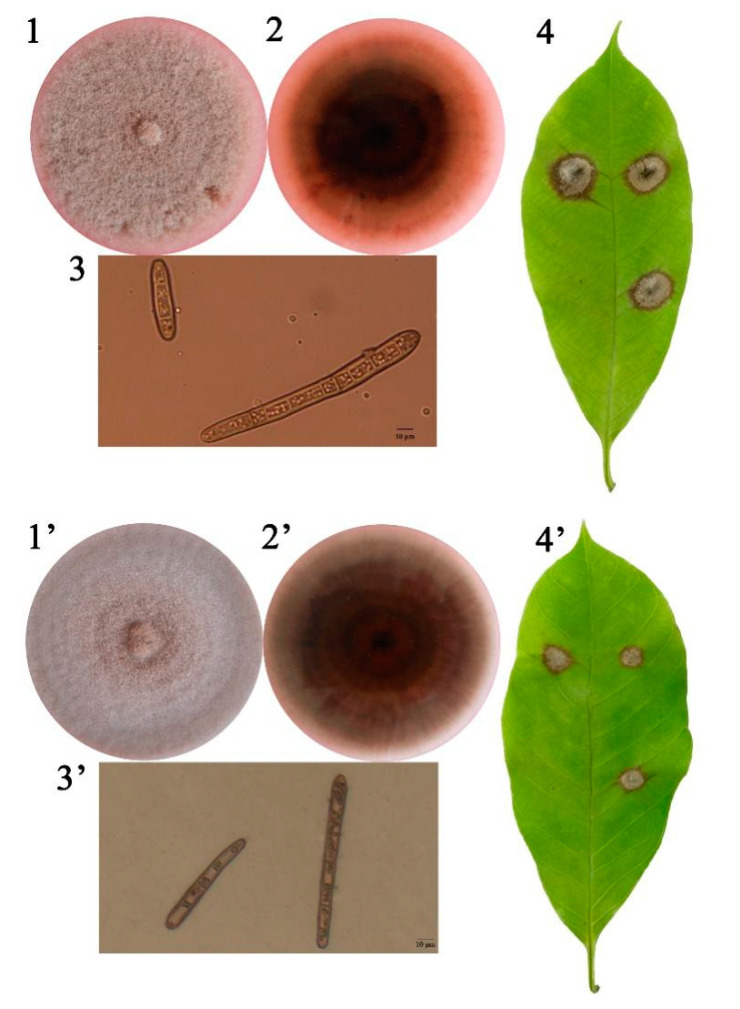
Symptoms of disease caused by *Corynespora cassiicola* YN49 and CC01 strains in *Hevea brasiliensis* clone Reyan7-33-97. The (**1**) and (**1′**): mycelium colony on PDA medium (front); (**2**) and (**2′**): mycelium colony on PDA medium (back); (**3**) and (**3′**): optical microscopy view of conidia in water; (**4**) and (**4′**): CLF symptoms on rubber tree clone Reyan7-33-97.

*C. cassiicola* YN49 and CC01 strains were separately isolated from rubber plantations in Hekou, the Yunnan province, and Yangjiang, the Guangdong province. Based on the sequence of the cassicolin-encoding gene, the YN49 strain belongs to Cas5 isolates and CC01 belongs to Cas2 isolates (Figure S1, Supplementary Materials File S7). YN49 and CC01 mycelia grown on PDA was fluffy and grey (Figure 1), similar to the CCP strain [16]. YN49 produced different pigments on PDA compared with other strains, and the PDA medium turned light orange as the mycelia aged (Figure 1). After 5 days of conidia inoculation, both YN49 and CC01 caused necrotic spots and typical darkening of the veins on the leaves of *Hevea brasiliensis* clone Reyan7-33-97, one of the main rubber tree cultivars in China. Compared with CC01, YN49 was more pathogenic toward Reyan7-33-97 (Figure 1).

### 3.2. General Genome Features and Annotation

Single-molecule real-time sequencing of long reads was conducted on a GridION X5 platform, and the genome of YN49 was sequenced with 115 × coverage, while the genome of CC01 was sequenced with 228 × coverage. CANU was used for de novo assembly of the sequencing data with Pilon-based continuous iteration correction using Illumina HiSeq4000 sequencing data, which generated 32 contigs with an N50 length of 2.51 Mb for the YN49 genome, and 33 contigs with an N50 length of 2.56 Mb for YN49. The genome size of YN49 (45.1 Mb) and CC01 (47.1 Mb) is slightly larger than that of HGCC (42.7 Mb), CCP (44.8 Mb) and UM591 (41.4 Mb). The GC content of YN49 (51.09%) and CC01 (50.96%) is lower than that of HGCC (51.78%), CCP (51.89%) and UM591 (52.47%; Table 1). The completeness of the genome assembly was assessed using BUSCO, which showed that 96.9% and 97.5% of the gene groups were correct assembled for the YN49 and CC01 scaffolds, respectively (Supplementary Materials File S1: Table S1). The YN49 genome contains 388 noncoding RNAs (ncRNAs) comprising 152 ribosomal RNAs (rRNAs), 44 small nuclear RNAs (snRNAs) and 192 transfer RNAs (tRNAs), while the CC01 genome contains fewer ncRNAs (338) comprising 79 rRNAs, 45 snRNAs and 214 tRNAs (Supplementary Materials File S1: Table S2). Furthermore, 8.18% of the YN49 genome and 9.18% of the CC01 genome are repetitive based on de novo and reference-based repeat analysis results (Supplementary Materials File S1: Table S3). A total of 14,504 protein-coding genes were predicted in the YN49 genome, for which 13,312 (91.78%) have at least one type of annotation from Cluster of Orthologous Groups of proteins (COG; 28.10% of genes), Gene Ontology (GO; 47.28%), Kyoto Encyclopedia of Genes and Genomes (KEGG; 16.91%), Non-Redundant Protein (NR; 91.71%) or Swiss-Prot (59.11%) databases. Additionally, 14,224 (82.61%) of 17,219 total predicted protein-coding genes have at least one type of annotation (COG 24.87%, GO 41.54%, KEGG 14.82%, NR 82.53%, Swiss-Prot 51.30%; Supplementary Materials File S1: Table S4).

### 3.3. Analysis of Orthologues and Phylogenetic Relationships between C. cassiicola YN49 and CC01 and Other Fungi

**Figure 2:**
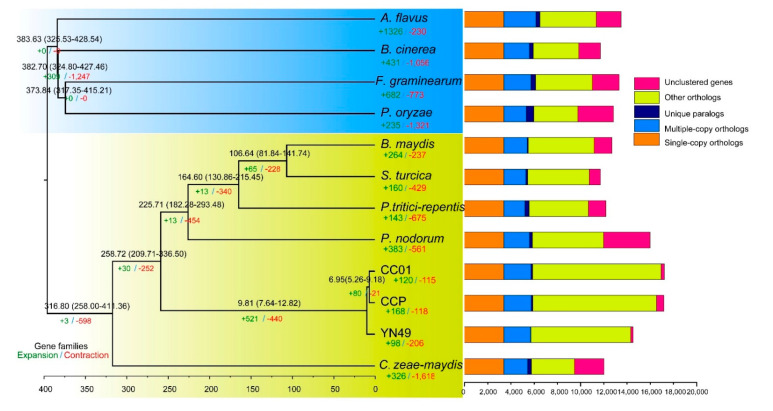
Phylogenetic relationships between *C. cassiicola* YN49 and CC01 strains and 10 other phytopathogenic fungi. Eurotiomycetes and Leotiomycetes fungi are indicated by a blue background, and yellow indicates Dothideomycetes fungi. Divergence times are labelled blue, gene family expansion and contraction are enumerated below the species names in green and red, respectively, and gene categories are shown on the right.

We clustered the annotated genes of *C. cassiicola* YN49 and CC01, and the other 10 phytopathogenic fungi, into gene families, including 3,388 single-copy genes, which were used for phylogenetic tree construction. A maximum likelihood phylogenetic tree was generated by the RaxML method, based on the GTRGAMMA model (Figure 2). The results revealed that *C. cassiicola* YN49 and CC01 are evolutionarily closely related to *C. cassiicola* CCP, a fungus isolated in the Philippines that causes CLF disease in rubber trees.

### 3.4. Gene Family Expansion and Contraction, and Positive Selection of Genes

**Figure 3:**
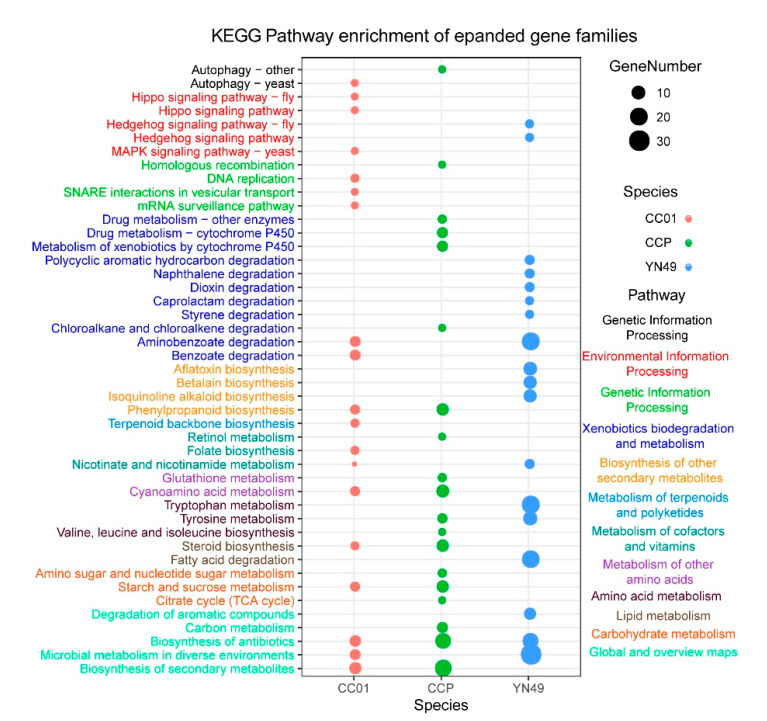
KEGG distribution of expanded gene families in *C. cassiicola* YN49, CC01 and CCP strains.

**Figure 4:**
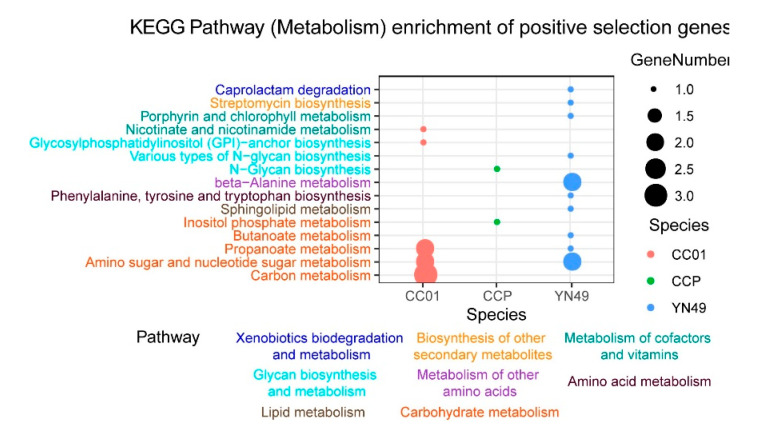
KEGG distribution (metabolic pathways) of positively selected genes in *C. cassiicola* YN49, CC01 and CCP strains.

The expansion and contraction of gene families is thought to be important in adaptive phenotypic diversification [41]. For plant pathogenetic fungi, continuous coevolution of host plants gives rise to constant selective pressure for the preservation of expanded gene families relevant to virulence and host-based nutrient usage [42]. Based on sequence homology, we identified 98 (432 genes), 120 (479 genes) and 168 (734 genes) gene families showing expansion in YN49, CC01 and CCP genomes, respectively (Figure 2 and Supplementary Materials File S2: Table S4). We also identified 206 (212 genes), 115 (178 genes) and 118 (131 genes) gene families showing contraction in YN49, CC01 and CCP genomes, respectively (Figure 2 and Supplementary Materials File S2: Table S5). KEGG pathway enrichment analysis of expanded and contracted gene families indicated that most of these gene families are associated with primary and secondary metabolism pathways, such as amino acid, fatty acid, terpenoid and aflatoxin metabolism (Figure 3 and Figure S2; Supplementary Materials File S2: Tables S1 and S2). In addition to the expanded and contracted gene families, genes showing positive selection commonly contribute to adaptive phenotypic evolution and adaptation. Herein, 61, 49 and 24 genes were identified as positively selected genes in YN49, CC01 and CCP genomes, respectively (Supplementary Materials File S2: Table S6). KEGG (metabolic pathway) enrichment analysis of these positively selected genes revealed that some KEGG pathways that were significantly enriched were related to carbon metabolism and amino acid metabolism (Figure 4 and Supplementary Materials File S2: Table S3).

### 3.5. Whole-Genome Synteny Comparisons between C. cassiicola YN49, CC01 and CCP

**Figure 5:**
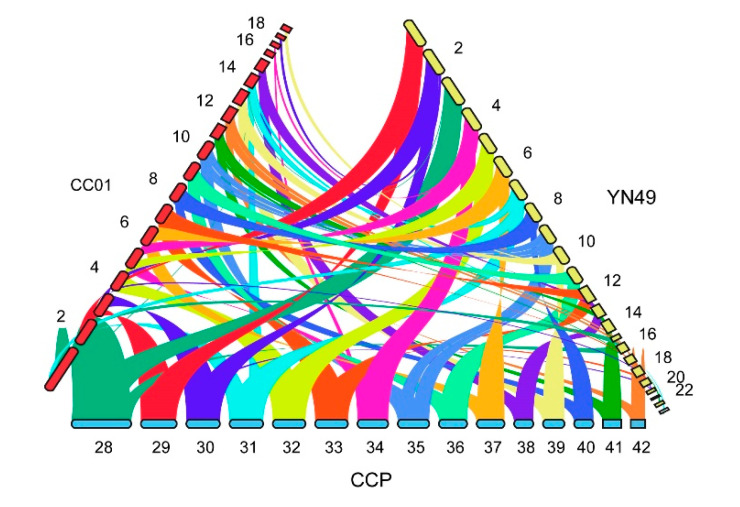
Synteny analysis of *C. cassiicola* YN49, CC01 and CCP strains. Phylogenomic analysis revealed that *C. cassiicola* YN49 and CC01 are evolutionarily closely related to *C. cassiicola* CCP. Therefore, we performed synteny comparison between these three species. The resulting synteny dot-plot shows macrosynteny between the three genomes, and high levels of sequence homology between strains. There are 19 contigs in YN49, 23 contigs in CC01 and 15 contigs in CCP with conserved syntenic blocks.

Phylogenomic analysis revealed that *C. cassiicola* YN49 and CC01 are evolutionarily closely related to *C. cassiicola* CCP. We therefore performed a synteny comparison between these three strains. The resulting synteny dot-plot displays macrosynteny between the three genomes, and high levels of sequence homology with each other; 19 contigs of YN49, 23 contigs of CC01 and 15 contigs of CCP have conserved syntenic blocks (Figure 5).

### 3.6. Secretome and Putative Pathogenicity Genes

**Figure 6:**
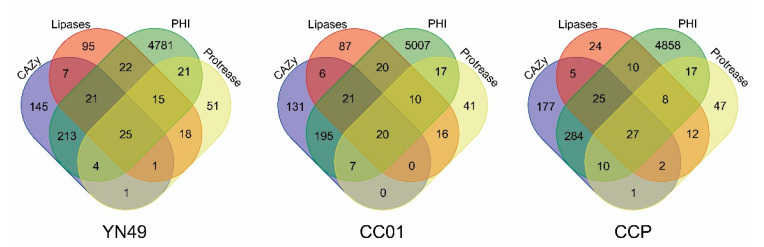
Venn diagram showing the overlap of different secreted gene categories with genes relevant to fungal pathogenicity.

The secretome of a plant pathogenetic fungus includes extracellular secreted proteins that are deployed to the host-pathogen interface during infection, including important virulence factors such as effector proteins for the manipulation of host cell dynamics and cell wall-degrading enzymes [43]. A secretome prediction pipeline for *C. cassiicola* [16] was implemented to predict the secretomes of *C. cassiicola* YN49, CC01 and CCP. A total of 1563 secreted proteins in the YN49 genome, 1474 in the CC01 genome, and 1534 in the CCP genome were predicted, accounting for 10.78%, 8.56% and 8.93% of their proteomes, respectively (Figure 6). To be a successful phytopathogen, *C. cassiicola* encodes an array of hydrolytic enzymes, including CAZymes (YN49 = 417; CC01 = 380; CCP = 531), proteases (YN49 = 136; CC01 = 111; CCP = 124), and lipases (YN49 = 204; CC01 = 180; CCP = 113) (Supplementary Materials File S3: Tables S1–S3). BLAST searching against the pathogen–host interactions (PHI) database [44] identified 5102, 5297 and 5239 putative pathogenicity genes in YN49, CC01 and CCP genomes, respectively (Figure 6 and Supplementary Materials File S4: Tables S1–S3). CAZymes and proteases are crucial for the degradation of the host plant cells, and to establish colonisation, while lipases also play an important role during the establishment of virulence [45]. The overlap of different secreted gene categories with genes relevant to fungal pathogenicity indicated that a large portion of secreted CAZymes (YN49 = 263, 63.07%; CC01 = 243, 63.95%; CCP = 346, 65.16%), proteases (YN49 = 83, 61.29%; CC01 = 71, 63.96%; CCP = 70, 56.45%), and lipases (YN49 = 65, 31.86%; CC01 = 54, 30.00%; CCP = 62, 54.87%) were relevant to pathogenicity (Figure 6).

### 3.7. Carbohydrate-Active Enzymes

Secreted carbohydrate degradation is an important component of fungal pathogenicity and virulence. Based on catalytic activity, CAZymes were further classified into auxiliary activities (AAs), carbohydrate esterases (CEs), glycoside hydrolases (GHs), glycosyl transferases (GTs), and polysaccharide lyases (PLs) [36]. We examined the CAZymes of *C. cassiicola* YN49, CC01 and CCP. Using the common CAZy annotation pipeline for the genomic analysis of fungi, we identified 417 putative secreted CAZymes falling into 83 CAZyme families in YN49, 380 falling into 79 families in CC01, and 531 falling into 101 families in CCP (Supplementary Materials File S3: Tables S1–S3). To overcome the barrier of the plant cell wall, phytopathogenic fungi produce enzymes that degrade cellulose, pectin and cutin, and that are capable of degrading cell wall polymers [19]. CAZymes involved in plant cell wall degradation, such as cellulose, hemicellulose pectin and cutin degradation, are listed in Table 2, according to classification by Chang [46] and Kubicek [19]. The results indicate that CCP possesses 223 secreted plant cell wall degradation-related CAZymes, 14.95% more than YN49 (194) and 21.19% more than CC01 (184).

### 3.8. Secondary Metabolite Gene Clusters

Phytotoxic SMs are crucial weapons that phytopathogenic fungi use to invade target plants, and many are made from polyketides, non-ribosomal peptides, terpenes and alkaloids [47]. Compared with the SMs mentioned above, beta-lactones are rarely found in plant pathogens. However, with improved biochemical knowledge and bioinformatic predictions, new beta-lactones with novel functions and related biosynthetic gene clusters are being identified in fungi [48,49]. Herein, AntiSMASH 5.1.2 (fungi view) was used to identify SM BGCs in the genomes of *C. cassiicola* YN49, CC01 and CCP, and all putative SM BGCs are listed in Supplementary Materials File S1: Table S5. As shown in Table 3, YN49 and CCP both have 57 SM BGCs, while CC01 has 62. All three strains share a similar number of BGCs of most types of SM, including NRPSs, PKSs (type I and III), and indole and terpene BGCs, but PKS/NRPS, PKS/indole and beta-lactone BGCs did differ somewhat between the target strains (Table 3). CC01 has 10 PKS/NRPS BGCs, nine more than YN49 and six more than CCP. There is a single beta-lactone BGC located in both YN49 and CCP genomes, but none in the CC01 genome. YN49 is the only strain possessing a single PKS/indole BGC (Table 3).

### 3.9. Phylogenomic Analysis of NRPS, PKS and PKS/NRPS Genes, and Domain Structure Analysis

**Figure 7:**
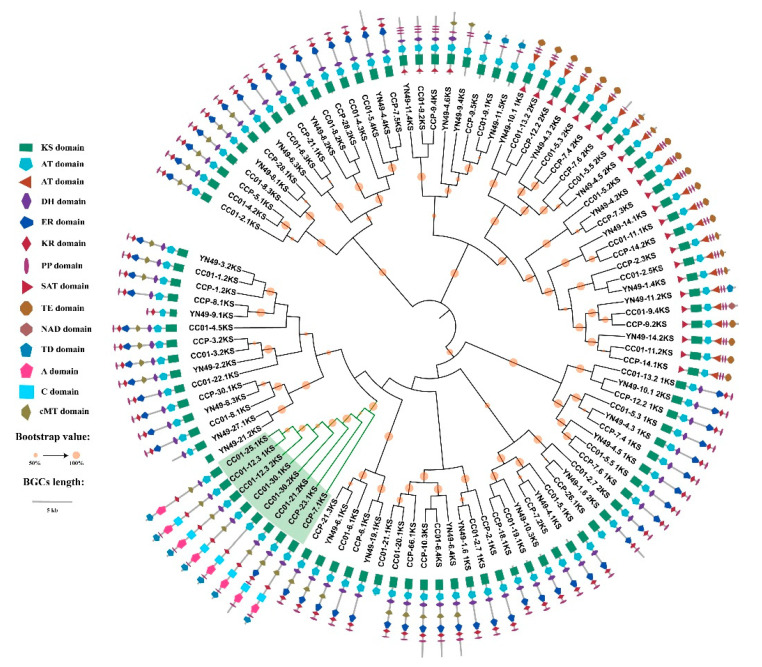
Phylogenetic and structure domain analyses of PKSs and PKS/NRPSs from *C. cassiicola* YN49, CC01 and CCP based on KS domain sequences. The PKS and PKS/NRPS domains were determined based on antiSMASH analysis. The ML tree was generated using MEGA 6 with the WAG model. Bootstrap support greater than 50% is shown on the branches. KS domain sequences used in this analysis are listed in Supplementary Materials File S5.

**Figure 8:**
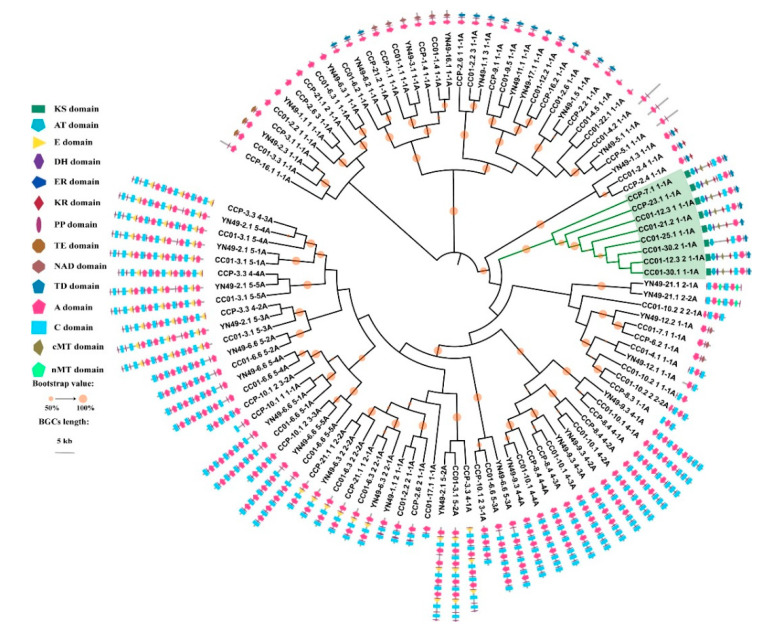
Phylogenetic and structural domain analysis of NRPSs and PKS/NRPSs from *C. cassiicola* YN49, CC01 and CCP based on A domain sequences. NRPSs and PKS/NRPSs domains were determined based on antiSMASH analysis. The ML tree was generated using MEGA 6 with the WAG model. Bootstrap support greater than 50% is shown on the branches. A domain sequences used in this analysis are listed in Supplementary Materials File S6.

In order to determine differences between the secondary metabolomes of *C. cassiicola* YN49, CC01 and CCP, we analysed the phylogenomic relationships of the NRPS, PKS, and PKS/NRPS BGCs identified in the three strains, based on the sequence of A and KS domains, which are relatively conserved in NRPSs and PKSs [50,51]. The phylogenomic relationships of A and KS domains indicated that most NRPSs and PKSs are conserved in these three strains, with many small clades containing three KS or A domains from three different strains (Figure 7 and Figure 8). Most NRPSs and PKSs in the clades share an identical or similar domain structure, but some have distinct structures that differ from those of the other two strains, such as CCP-26.1KS (Figure 7) and CCP-10.1 2 3-1A (Figure 8). Compared with NRPSs and PKSs, PKS/NRPS BGCs displayed larger differences in these three strains. Although YN49 has one PKS/NRPS gene cluster containing one PKS gene and one NRPS gene, there is no PKS/NRPS gene in the YN49 genome. CC01 and CCP have six and two PKS/NRPS genes, respectively.

### 3.10. Synteny Analysis of PKS/NRPS Gene Clusters

**Figure 9:**
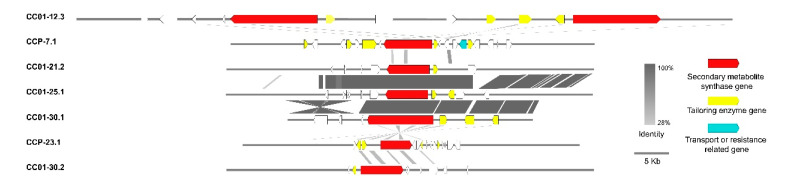
Synteny analysis of PKS/NRPS gene clusters in *C. cassiicola* CC01 and CCP.

Our results showed that a number of PKS/NRPS clusters differ significantly between *C. cassiicola* YN49, CC01 and CCP (Table 3). Additionally, phylogenomic relationship analysis indicated that the PKS/NRPS genes shared high homology, and could be clustered into one clade based on the sequences of the A or KS domains (Figure 7 and Figure 8). An intact SM BGC contains genes involved in product modification, transport, and transcription regulation, but not backbone synthesis genes whose enzymatic products produce a core metabolite [52]. To analyse the differences between PKS/NRPS clusters in target *C. cassiicola* strains, synteny of the PKS/NRPS clusters was analysed. Among the seven tested clusters, only three (CC01-21.1, CC01-25.1 and CC01-30.1) displayed good synteny. CCP-23.1, CC01-30.2 and CC01-30.1 share some synteny, but CC01-12.3 and CCP-7.1 have almost no synteny with other analysed clusters (Figure 9). These results indicate large differences among the PKS/NRPS gene clusters in target *C. cassiicola* strains, which suggests that secondary metabolism may vary between *C. cassiicola* strains.

## 4. Discussion

*C. cassiicola* strains have been isolated from different plants with various life styles, including endophyte, saprophyte, and many necrotrophic pathogens [16,53,54,55]. Rubber tree CLF disease, caused by *C. cassiicola*, is a devastating leaf disease affecting rubber plantations in many countries in Asia and Africa, and it also threatens rubber production in China [14,16,56]. *C. cassiicola* isolates show high genetic diversity, and virulence profiles vary significantly between rubber tree cultivars [14]. Based on the amino acid sequence of the phytotoxin cassiicolin, *C. cassiicola* is clustered into the following seven types: Cas1–6 and Cas0. Cas2 isolates have only been found in China [15]. Except cassiicolin, little is known about the pathogenesis and associated pathogenic factors of *C. cassiicola*. Although 41 *C. cassiicola* genome assemblies are available in the NCBI database (https://www.ncbi.nlm.nih.gov/genome/browse/#!/eukaryotes/31373/ accessed on 13 October 2020), most assemblies are not annotated, and are composed of thousands of contigs. CCP is the only annotated assembly, but it also contains hundreds of contigs [16]. In the present study, we determined two high-quality gapless genome sequences, acquired by single-molecule real-time sequencing, of two *C. cassiicola* strains isolated from rubber trees in China with different virulence, including one Cas2 isolate. To decipher the genomic basis underlying the pathogenesis of CLF disease, we performed a comparative analysis of genomic data between Chinese strains and the highly virulent Philippine strain CCP.

Utilisation of host-based nutrition is critical for fungal parasitism, and gene families can contract or expand to adapt to distinct host-based nutrients [57]. For plant pathogenetic fungi, advantageous substitutions that enhance the capacity to infect hosts or adapt to new environments are likely to be rapidly fixed in the population [58]. Rubber trees are traditionally planted between 15° N and 10° S, but China has 1.13 million hectares of rubber plantations between 18° N and 24° N. Due to the higher latitude, cold weather is the most serious threat to rubber plantations in China. Thus, cold-resistant rubber clones have been developed and are widely cultivated in Chinese rubber tree plantations [59,60]. Different rubber tree cultivars result in different *C. cassiicola* CFL strains, including Cas2 type isolates that infect pepper, cucumber and papaya worldwide, but infect rubber trees only in China. The virulence profiles of *C. cassiicola* vary significantly depending on rubber tree cultivars, and also display geographical specialisation [14,56]. There are the following two hypotheses explaining host and geographical specialisation of *C. cassiicola*: the coevolution of isolates with different rubber cultivars, and host switching from other plants. Based on our results, the Chinese Cas2 isolate CC01 appears to have diverged phylogenetically from CCP approximately 6.95 (5.29–9.18) million years ago (MYA), and the Chinese Cas5 isolate YN49 diverged ~9.81 (7.64–12.82) MYA (Figure 2). Since rubber trees have only been grown in China for less than 100 years, CC01 and YN49 have presumably transferred from other host plants to rubber trees. We also found that the most expanded and contracted gene families in the genomes of the two Chinese *C. cassiicola* CFL strains and the Philippine strain are related to metabolic pathways (Figure 3 and Supplementary Materials, Table S4), and a large proportion of positively selected genes, which reflect the evolutionary pressure imposed by natural selection, in these three strains are metabolism-related (Figure 4 and Additional File 2: Table S6), including genes involved in carbohydrate metabolism and secondary metabolism. The diversity of metabolism-related genes allows *C. cassiicola* to adapt to different rubber cultivars, and this may lead to differences in pathogenesis and/or pathogenic factors, as YN49 and CC01 showed different virulence (Figure 1).

Plant pathogenic fungi can secrete a series of proteins that are deployed to the host–pathogen interface during infection, including enzymes interacting with plant substrates (CAZymes, peptidases and lipases), together with proteins of unknown function [61]. Necrotrophic and hemibiotrophic plant pathogenic fungi usually secrete a larger number of enzymes that are more important for host invasion than biotrophs [62,63]. Our results showed that all three tested *C. cassiicola* strains possess a large number of secreted protein-coding genes in their genomes. In agreement with expanded and contracted gene families results, the number of secreted protein-coding genes differed between all three tested *C. cassiicola* strains. The Cas1 isolate (CCP) has more secreted protein-encoding genes than the Cas2 isolate (CC01) and the Cas5 isolate (YN49), consistent with previous reports [16] and phenotype results (Figure 1).

CAZymes are important for carbon acquisition and metabolism in fungi, and CWDEs are used by phytopathogenic fungi as powerful weapons to penetrate and degrade the plant cell wall [64]. The Cas2 isolate (CC01) and the Cas5 isolate (YN49) were found to contain fewer CWDEs than the Cas1 isolate (CCP) in the present study, especially the Cas2 isolate (Table 2). The most obvious difference was the number of pectin- and cutin-degrading enzymes, and GH5, which is related to cellulose degradation (Table 2). Pectinolytic enzymes are important in pathogenesis as potential virulence factors, especially in phytopathogens affecting dicots, since the pectin content of the cell wall (30%) is much higher than that of monocots (10%) [65,66]. Other pathogenetic fungi affecting rubber trees, such as *Colletotrichum* spp., also secrete pectin lyases to invade rubber leaves [67,68]. CCP encodes many more secreted pectin lyases than YN49 and CC01, such as GH28, PL1, and three other families, and CC01 possesses the fewest pectinolytic enzyme-encoding genes. This suggests that pectin degradation ability varies between different *C. cassiicola* strains, and Cas1 isolates might degrade pectin components more effectively than Cas2 and Cas5 isolates, this could partially explain the pathogenicity difference between different *C. cassiicola* strains.

Rubber tree leaves are covered with a thick, waxy cuticle, and the thickness is related to pathogen resistance [18,69]. The cuticle consists a matrix of mid-chain hydroxy and/or epoxy C16 and/or C18 fatty acid monomers and waxes, including numerous very-long-chain fatty acids (VLCFAs; C20–C40). Both the cuticle and the waxes cover the surface of leaves to prevent water loss and invasion of pathogens [70]. To breach these hydrophobic layers, many phytopathogenic fungi secrete cutinases and lipases in the early stages of infection, as demonstrated for *Valsa mali*, *Blumeria graminis* and *F. graminearum* [45,47,71]. One CCP strain was found to encode 11 putative cutinases, more than are present in hemibiotrophs, aprotrophs and ectomycorrhizal fungi [16]. Herein, we analysed the distribution of secreted cutinases between different cassicolin toxin classes, and the results indicated that Cas1 isolates possess more secreted cutinase-encoding genes than Cas2 and Cas5 isolates, with Cas2 isolates containing the fewest. The trend in the number of secreted lipase-encoding genes differed from the results of cutinase analysis; CCP encodes fewer secreted lipase genes than both YN49 and CC01. In order further evaluate the lipid degradation ability, we combined the results of secreted lipase and PHI analyses, and found that CCP possesses more pathogenicity-relevant lipases than CC01, but fewer than YN49 (Figure 3). Thus, Cas1 isolates (CCP) and Cas5 isolates (YN49) appear to encode more putative cuticle and wax degradation enzymes than Cas2 isolates (CC01), and this might be one of the reasons explain why CC01 showed less virulence than YN49 (Figure 1).

Phytotoxins (PTs) are largely represented by low-molecular-weight SMs that disrupt vital activities of plant cells and/or cause death at concentrations below 10 mM [72]. Cassiicolin is the only phytotoxin of *C. cassiicola* characterised to date, and the cassiicolin gene has only been detected in 47% of isolates. Based on the presence of the cassiicolin toxin and its amino acid sequence, *C. cassiicola* strains are divided into seven categories (Cas1–6 and Cas0) [14]. Since *C. cassiicola* shows high genetic diversity and little correlation between genetic clades and the traits of strains, many studies have attempted to connect cassiicolin types with pathogenicity and geographical locations. Most such studies have been carried out on rubber trees, and the results vary between different cultivars. The only thing in common is that Cas1 isolates, especially CCP strains, are more virulent toward some specific cultivars [8,14,73].

In our previous study, we analysed the pathogenicity of three *C. cassiicola* isolates of three different toxin classes (Cas2, Cas5 and Cas0), which are the dominant populations in Chinese rubber plantations, toward four different rubber tree cultivars. All three isolates showed differences in pathogenicity toward the four clones, and there were no obvious trends [74]. In the present work, YN49 showed stronger virulence than CC01 toward *Hevea brasiliensis* clone Reyan7-33-97 (Figure 1). Comparative genomic results showed that CCP contains many more CWDEs than YN49 and CC01, and CC01 has the fewest such enzymes. Thus, we speculate that the cassicolin toxin is not the only pathogenic factor affecting the virulence of *C. cassiicola* toward rubber trees, and secreted CWDEs also play an important role during pathogenesis, particularly in the early stages of infection. Secreted CWDEs are potent weapons used by *C. cassiicola* to breach the cell wall barrier, and assist the release of cassicolin and other pathogenic factors. Thus, a thick cuticle and wax layer covering rubber tree leaves, and other enhanced cell wall structures, may effectively prevent the invasion of *C. cassiicola* strains lacking CWDEs, but may be unable to block the penetration of *C. cassiicola* strains containing numerous CWDEs. Since the resistance of rubber tree leaves and CWDEs of different *C. cassiicola* strains remain unclear, the results of pathogenicity tests on the CFL strains were somewhat difficult to interpret. This may partially explain why different rubber tree clones show differences in susceptibility to CLF disease. For example, GT1 is resistant in Africa but highly sensitive in Thailand [8].

Numerous *C. cassiicola* strains do not encode the cassicolin toxin, and some can still cause CFL disease in rubber trees in many areas, including China, India and Thailand [8,62]. Similarly, serval Cas0 isolates were found to infect cucumber [15]. These findings indicate that *C. cassiicola* contains other pathogenic factors in addition to the cassicolin toxin. Phytopathogenic fungi, especially filamentous fungi, usually contain dozens of SM BGCs encoding a diverse array of small molecules that function as phytotoxins, antibiotics and pigments [24,52]. Herein, we identified ~60 SM BGCs in three different *C. cassiicola* strains, many more than the average for phytopathogenic fungi, consistent with previous studies, indicating that *C. cassiicola* possesses the genomic basis for the synthesis of various SMs [16,75]. Based on the genomes of 66 different *Aspergillus fumigatus* strains, Lind et al. (2017) summarised five general types of variation in SM BGCs within a fungal species, and revealed diversity and discontinuity in the distributions of SM BGCs, including between strains sharing high levels of synteny [52]. Although the total number of SM BGCs is similar in the three analysed *C. cassiicola* strains, they have significant differences in the number of genes encoding beta-lactone, PKS/indole and NRPS/indole SM BGCs, and especially PKS/NRPS BGCs (Table 3). PKS/indole and NRPS/indole BGCs are only present in YN49 and CC01, and beta-lactone BGCs are not present in CC01, indicating whole-gene cluster polymorphisms in *C. cassiicola* for SM BGCs. Further phylogenomic and domain structure analyses showed that although most PKSs and NRPSs could be clustered into one clade with high bootstrap value support, some parts had distinct domain structures, such as the PKS of the CCP-7.5 cluster and the YN49-11.2 cluster, and the NRPS of the CCP-10.1 cluster (Figure 7 and Figure 8). This is consistent with single nucleotide polymorphisms (SNPs) and short indel polymorphisms. All the PKS/NRPSs could be clustered into one clade based on A or KS domain sequences, suggesting that PKS/NRPSs are relatively highly conserved between *C. cassiicola* strains (Figure 7 and Figure 8). However, synteny analysis of clusters containing PKS/NRPSs gave diverse results; only three clusters showed synteny, while the rest shared almost no synteny (Figure 9). This indicates gene content polymorphisms in *C. cassiicola* SM BGCs, even between clusters with similar core genes. *C. cassiicola* SM BGC polymorphisms likely correlate with differences in SMs between strains, and this may be one of the reasons why *C. cassiicola* displays complex pathotypic diversity.

## 5. Conclusions

This study determined high-quality whole-genome sequences of two Chinese isolates (YN49 and CC01) of *C. cassiicola* that cause CLF disease of rubber trees. Comparative genomics of gene families in these two stains and the virulent CCP strain from the Philippines showed that *C. cassiicola* strains with different geographic origins have experienced different selective pressures, resulting in differences in metabolism-related gene families. Secreted protein analysis indicates that the number of secreted CWDEs is correlated with pathogenesis, and the structural resistance of rubber tree leaves may also influence the pathogenicity of CFL strains. *C. cassiicola* strains encode numerous SM BGCs, and there is significant diversity and discontinuity in the distribution of SM BGCs between *C. cassiicola* strains. This implies SM polymorphisms between *C. cassiicola* strains, which may play an important role in pathogenic progress. These findings form the basis for further experimental studies on the pathogenesis of rubber tree CLF disease.

## Figures and Tables

**Table 1:** Genome characteristics and assembly features of *C. cassiicola* isolates YN49, CC01, HGCC, CCP and UM591.

Features	YN49	CC01	HGCC	CCP	UM591
Total genome size (Mb)	45.1	47.1	42.7	44.8	41.4
Genome coverage	115×	228×	184×	123×	118×
G + C content (%)	51.09	50.96	51.78	51.89	52.47
Number of contigs	32	33	1032	644	1941
Contig N50 (bp)	2,523,863	2,573,660	/	211,447	44,332
Protein-coding genes	14,504	17,219	14,631	17,167	14,744

**Table 2:** CAZymes involved in plant cell wall degradation.

Substrates	Family	YN49	CC01	CCP
Cellulose	AA9	25	28	27
	GH1	2	1	3
	GH12	2	2	2
	GH3	13	12	13
	GH45	1	2	2
	GH5	31	27	37
	GH6	3	2	2
	GH7	5	5	5
Hemicellulose	CE1	8	8	9
	GH10	3	4	4
	GH11	4	4	4
	GH115	2	2	2
	GH27	2	3	4
	GH29	1	0	1
	GH30	2	2	2
	GH35	3	3	4
	GH36	1	1	1
	GH39	1	1	1
	GH43	25	21	24
	GH51	0	0	1
	GH53	1	1	1
	GH67	1	1	1
	GH93	3	3	2
Pectin	CE12	4	2	5
	CE8	4	3	4
	GH105	5	4	6
	GH28	7	9	10
	GH78	2	5	4
	PL1	11	12	17
	PL3	11	8	12
	PL4	4	2	4
	PL9	2	2	2
Cutin	CE5	5	4	7
Total number		194	184	223

**Table 3:** Secondary metabolite biosynthetic gene clusters in *C. cassiicola* YN49, CC01 and CCP.

SM Cluster Types	YN49	CC01	CCP
NRPS	16	15	15
PKS (Type I)	26	25	26
PKS (Type III)	1	1	1
PKS/NRPS	1	9	4
PKS/Indole	1	0	0
NRPS/Indole	0	1	0
Indole	1	1	1
Terpene	10	10	9
Beta-lactone	1	0	1
Total	57	62	57

## Data Availability

The whole-genome sequencing datasets from this study have been submitted to the BioProject database of the National Center for Biotechnology Information (NCBI) (https://www.ncbi.nlm.nih.gov/ accessed on 13 October 2020) under accession number PRJNA687613 and PRJNA687612. All sequencing raw data were uploaded to the NCBI Sequence Read Archive (http://www.ncbi.nlm.nih.gov/sra accessed on 13 October 2020) database under the GenBank accession numbers SRR13318044 and SRR13316927.

## Supplementary Materials

The following are available online at https://www.mdpi.com/article/10.3390/jof7060485/s1, File S1: Table S1: BUSCO analysis of *C. cassiicola* isolates YN49 and CC01 scaffolds assembly; Table S2: RNA statistics of *C. cassiicola* isolates YN49 and CC01; Table S3: Repeative sequence statistic of *C. cassiicola* isolates YN49 and CC01; Table S4: Gene annotation statistic of *C. cassiicola* isolates YN49 and CC01; Table S5: SM Clusters coordinates of *C. cassiicola* isolates YN49, CC01 and CCP scaffolds assembly. File S2: Table S1: KEGG distribution of expanded gene families of *C. cassiicola* YN49, CC01 and CCP; Table S2: KEGG distribution of contracted gene families of *C. cassiicola* YN49, CC01 and CCP; Table S3: KEGG distribution of positive selection genes of *C. cassiicola* YN49, CC01 and CCP; Table S4: Expanded gene families of *C. cassiicola* YN49, CC01 and CCP; Table S5: Contracted gene families of *C. cassiicola* YN49, CC01 and CCP; Table S6: Positive selection genes of *C. cassiicola* YN49, CC01 and CCP. File S3: Table S1: Secreted Cazymes of *C. cassiicola* YN49, CC01 and CCP; Table S2: Secreted protease of *C. cassiicola* YN49, CC01 and CCP; Table S3: Secreted lipases of *C. cassiicola* YN49, CC01 and CCP. File S4: Table S1: Genes relevant to fungal pathogenicity of *C. cassiicola* YN49; Table S2: Genes relevant to fungal pathogenicity of *C. cassiicola* CC01; Table S3: Genes relevant to fungal pathogenicity of *C. cassiicola* CCP. File S5: Sequences of KS domains used in this study. File S6: Sequences of A domains used in this study. File S7: *C. cassiicola* starins and their Cas gene sequences used in this study. Figure S1: Phylogenic analysis of *C. cassiicola* starins with Cas gene sequence; Figure S2: KEGG pathway enrichment of contracted gene families.
