# Supplementary material for: Genomic Characteristics and Comparative Genomics Analysis of Two Chinese Corynespora cassiicola Strains Causing Corynespora Leaf Fall (CLF) Disease
Source: J Fungi (Basel). 2021 Jun 16;7(6):485. doi: 10.3390/jof7060485 (PMC8235470; doi:10.3390/jof7060485)
Supplement: Supplementary file 1 [file jof-07-00485-s001.zip › jof-1246405-supplementary/Additionalfile 7.pdf]

Table Strains and gene information

Strain	Host	Geographical origin	Cassiicolin isoform	GeneBank
YN49	*Hevea brasiliensis*	China	Cas5	MW436470
Cc01	*Hevea brasiliensis*	China	Cas2	MW436471
CCAM2	*Hevea brasiliensis*	Cameroon	Cas1	JF915149
CGHA1	*Hevea brasiliensis*	Ghana	Cas1	JF915152
GSO2	*Vernonia cinerea*	Brazil	Cas2	JF915166
HGD01	*Hevea brasiliensis*	China	Cas2	KU561095
ATI13	*Glycine max*	Brazil	Cas2	JF915168
JMP2020	*Lantana camara*	Brazil	Cas2	JF915167
IA	*Cucumis sativus*	Brazil	Cas2	JF915163
CMENY	*Cucumis sativus*	Brazil	Cas2	JF915161
CBS	*Cucumis sativus*	Brazil	Cas2	JF915160
ATI17	*Gossypium hirsutum*	Brazil	Cas2	JF915159
777AA	*Glycine max*	Brazil	Cas2	JF915157
493AA	*Glycine max*	Brazil	Cas2	JF915156
ATI11	*Cucumis sativus*	Brazil	Cas2	JF915158
EDIG	*Cucumis sativus*	Brazil	Cas2	JF915162
PB	*Cucumis sativus*	Brazil	Cas2	JF915165
RUD	Glycine max	Brazil	Cas2	JF915164
E70	*Hevea brasiliensis*	Brazil	Cas3	JF915169
E78	*Hevea brasiliensis*	Brazil	Cas3	JF915170
E79	*Hevea brasiliensis*	Brazil	Cas4	JF915171
E139	*Hevea brasiliensis*	Brazil	Cas4	JF915172
HHN10	*Hevea brasiliensis*	China	Cas5	KU561094
CSB05A	*Hevea brasiliensis*	Malaysia	Cas5	KM873321
CSB16	*Hevea brasiliensis*	Malaysia	Cas5	KM873324
CSB5	*Hevea brasiliensis*	Malaysia	Cas5	JF915177
C68-6	*Hevea brasiliensis*	Malaysia	Cas5	KM873315
CSB05B	*Hevea brasiliensis*	Malaysia	Cas5	KM873322
CSB1	*Hevea brasiliensis*	Malaysia	Cas5	JF915175
CSB11	*Hevea brasiliensis*	Malaysia	Cas5	KM873323
CSB3	*Hevea brasiliensis*	Malaysia	Cas5	KM873325
CSB4	*Hevea brasiliensis*	Malaysia	Cas5	JF915176
CSB6	*Hevea brasiliensis*	Malaysia	Cas5	JF915178
CKT05F	*Hevea brasiliensis*	Malaysia	Cas5	KM873319
CSR12	*Hevea brasiliensis*	Sri Lanka	Cas5	JF915180
CKT05C	*Hevea brasiliensis*	Malaysia	Cas5	KM873317
C68-8	*Hevea brasiliensis*	Malaysia	Cas5	KM873316
CKT1	*Hevea brasiliensis*	Malaysia	Cas5	KM873320
CSB16R1	*Hevea brasiliensis*	Malaysia	Cas5	JF915179
SS1	*Hevea brasiliensis*	Malaysia	Cas5	JF915173
ATI13	*Glycine max*	Brazil	Cas6	JF915181
RUD	*Glycine max*	Brazil	Cas6	JF915183

>CCAM2
TACTTTTCTTAAGTATCATCAATTCCAAATCTTGAAAATCTGTCCTATACATTTGCTACACAATGAAATA
TCTCCCTATCCTCATCTCTGCTTTTGTAGCAGCCGTTGCTGCAGCCCCGCAAGATCCGTCTGCTGTGGCA
CCTGTACTCCCTAGACAGACTTGCGTACGTCTTGACTCACAAAAGCGCGATTAGAAAAGAGATGAAAAGG
TTGTAGCTAATAATTAGTAAAGGTAAGCTGTGTCAATTTCGGCAATGGGTTTTGTGGCGATAACTGTGGT
AATTCTTGGGCTGTAAGTGCTTTGTCTGTTTCTCAAATCTAAAGCTAATTTGCATGCAGTGTTCGGGATG
TTAACTTTGGTAGCATTCCAAAGAATTGCGGCCCACAAGATAGTGTAGCTTGAATATTTGAGCTAGCTGC
CGCTACAAGTCTATAGGATGGCAACCTTAGCTACCTATACGCAAGTCTCTTGCTCCTAGGTTGTTTAGAC
CGATTTAAATGTTATGTGTATATAATTAGCGCTACATATACACCACACTCCTTTACATACACAAATTTAT
ACTCTTACGGATCC
>CGHA1
TACTTTTCTTAAGTATCATCAATTCCAAATCTTGAAAATCTGTCCTATACATTTGCTACACAATGAAATA
TCTCCCTATCCTCATCTCTGCTTTTGTAGCAGCCGTTGCTGCAGCCCCGCAAGATCCGTCTGCTGTGGCA
CCTGTACTCCCTAGACAGACTTGCGTACGTCTTGACTCACAAAAGCGCGATTAGAAAAGAGATGAAAAGG
TTGTAGCTAATAATTAGTAAAGGTAAGCTGTGTCAATTTCGGCAATGGGTTTTGTGGCGATAACTGTGGT
AATTCTTGGGCTGTAAGTGCTTTGTCTGTTTCTCAAATCTAAAGCTAATTTGCATGCAGTGTTCGGGATG
TTAACTTTGGTAGCATTCCAAAGAATTGCGGCCCACAAGATAGTGTAGCTTGAATATTTGAGCTAGCTGC
CGCTACAAGTCTATAGGATGGCAACCTTAGCTACCTATACGCAAGTCTCTTGCTCCTAGGTTGTTTAGAC
CGATTTAAATGTTATGTGTATATAATTAGCGCTACATATACACCACACTCCTTTACATACACAAATTTAT
ACTCTTACGGATCC
> E70
AAATCTTCTAAACATTGTCGATTCCAGATCTTTCAAACCTGTCCCATACATTTGCTACATAATGAAATAC
CTCCCTATCCTCATCTCGGCTTTCGTAGCAGCCATTACTGCGGCTCCACAAGATCCGTCTGCTGTGGCAC
CTTTGCTCCCTAGACAGTCTTGCGTACGTCTTGACTCATCGAATGACAGCCAGGAAATGAAACAACATGT
AGCTAATAGTAGATAAAGGTATCGTGTGTCAATTTTGGCAATGGGTTCTGTGGCGATAACTGTGGCAATT
CCTGGGCTGTAAGTATTTAACATCTGTTTCTCAAATCTAAAGCTAATTTGCATACAGTGTTCGGGCTGTT
AGCTTAAGTAGCATTCTGGAGAATTGTGGTCCACAAGACAGTCTAGCTTGAATACTATAGCTAGCTGTTG
CTACAAGTCTACAGGGTGGCAGCACTGATTGCCTATATAGGCAATTTGTTGCTACCAAGTATTCTGGACT
GTTTCTCTCGTTACTTGTATATACTTGGCGCTATATATACACCACACTTATTTACATACCGCAAATCCAA
ATCCTTGCAGATCC
> E78
AAATCTTCTAAACATTGTCGATTCCAGATCTTTCAAACCTGTCCCATACATTTGCTACATAATGAAATAC
CTCCCTATCCTCATCTCGGCTTTCGTAGCAGCCATTACTGCGGCTCCACAAGATCCGTCTGCTGTGGCAC
CTTTGCTCCCTAGACAGTCTTGCGTACGTCTTGACTCATCGAATGACAGCCAGGAAATGAAACAACATGT
AGCTAATAGTAGATAAAGGTATCGTGTGTCAATTTTGGCAATGGGTTCTGTGGCGATAACTGTGGCAATT
CCTGGGCTGTAAGTATTTAACATCTGTTTCTCAAAGCTAAAGCTAATTTGCATACAGTGTTCGGGCTGTT
AGCTTAAGTAGCATTCTGGAGAATTGTGGTCCACAAGACAGTCTAGCTTGAATAGTATAGCTAGCTGTTG
CTACAAGTCTACAGGGTGGCAGTACTGATTGCCTATATAGGCAATTTGTTGCTACCAAGTATTCTGGACT
GTTTCTCTCGTTACTTGTATATACTTGGCGCTATATATACACCACACTTATTTACATACCGCAAATCCAA
ATCCTTGCAGATCC
>E79
AAATCTTCTAAACATTGTCGATTCCAGATCTTTCAAACCTGTCCCATACATTTGCTACATAATGAAATAC
CTCCCTATCCTCATCTCGGCTTTCGTAGCAGCCGTTACTGCGGCTCCACAAGATCCGTCTGCTGTGGCAC
CTTTGCTCCCTAGACAGTCTTGCGTACGTCTTGACTCATCGAATGACAGCCAGGAAATGAAACAACATGT

AGCTAATAGTAGATAAAGGTATCGTGTGTCAATTTTGGCAATGGGTTCTGTGGCGATAACTGTGGCAATT
CCTGGGCTGTAAGTATTTAACATCTGTTTCTCAAATCTAAAGCTAATTTGCATACAGTGTTCGGGCTGTT
AGCTTAAGTAGTATTCTGGAGAATTGTGGTCCACAAGACAGTCTAGCTTGAATACTATAGCTAGCTGTTG
CTACAAGTCTACAGGGTGGCAGTACTGATTGCCTATATAGGCAAATTTGTTGCTACCAAGTATTCTGGAC
TGTTTCTCTCGTTACTTGTATATACTTGGCGCTATATATACACCACACTCATTTACATACCGCAAATCCA
AATCCTTGCAGATCC

>E139

AAATCTTCTAAACATTGTCGATTCCAGATCTTTCAAACCTGTCCCATACATTTGCTACATAATGAAATAC
CTCCCTATCCTCATCTCGGCTTTCGTAGCAGCCGTTACTGCGGCTCCACAAGATCCGTCTGCTGTGGCAC
CTTTGCTCCCTAGACAGTCTTGCGTACGTCTTGACTCATCGAATGACAGCCAGGAAATGAAACAACATGT
AGCTAATAGTAGATAAAGGTATCGTGTGTCAATTTTGGCAATGGGTTCTGTGGCGATAACTGTGGCAATT
CCTGGGCTGTAAGTATTTAACATCTGTTTCTCAAATCTAAAGCTAATTTGCATACAGTGTTCGGGCTGTT
AGCTTAAGTAGTATTCTGGAGAATTGTGGTCCACAAGACAGTCTAGCTTGAATACTATAGCTAGCTGTTG
CTACAAGTCTACAGGGTGGCAGTACTGATTGCCTATATAGGCAAATTTGTTGCTACCAAGTATTCTGGAC
TGTTTCTCTCGTTACTTGTATATACTTGGCGCTATATATACACCACACTCATTTACATACCGCAAATCCA
AATCCTTGCAGATCC

>ATI13*(Cas6)

TGCGCTTCTTAAGTACCATCAATTCCAATTCTTTCAAACCTGTCCTATACATTTGCTACACAATGAAATA
TTTCCCTATCCTCATCTCTGCTTTTGTAGCAGCCGTTGCTGCAGCCCCGCAGGGTCCGTCTGCTGTGGCA
GCAGCTGTCCTTCCTAGACAGACTTGCGTACGTCTGGAATTATTAAAGCACAATTAGAAAAAAAGAAACA
ATATGTAGCTAATACTTAAATAAAGGCTTTTTGTGAATACTTTGGCAATGGATATTGCGGAAACACTTGT
ACTGGTGATTCTTGGGCTGTAAGTATTTAATTTCTGTTTCTTGAATCTAAAGCTGATTTGCATACAGTGT
TCGAATTGTTAACTTAGTTAGCGTTCTAGAGAATTGCGGCCCACAAGATGGTCTAGCTTGAATGTTTGAG
CTAGCTGTTGCTGCCAGTCTATAGGATGGCAACCTTAGCTACCTATACGCAAGTCTCTTGCTCCTGAGTC
ATCTAGATCGATTTACATGTTATGTGTATATACTTAGCGCTACATATACACCACACTCCTTTACATACAC
AAATTCATATTCTTACGGATCC

>RUD

TGCGCTTCTTAAGTACCATCAATTCCAATTCTTTCAAACCTGTCCTATACATTTGCTACACAATGAAATA
TTTCCCTATCCTCATCTCTGCTTTTGTAGCAGCCGTTGCTGCAGCCCCGCAGGGTCCGTCTGCTGTGGCA
GCAGCTGTCCTTCCTAGACAGACTTGCGTACGTCTGGAATTATTAAAGCACAATTAGAAAAAAAGAAACA
ATATGTAGCTAATACTTAAATAAAGGCTTTTTGTGAATACTTTGGCAATGGATATTGCGGAAACACTTGT
ACTGGTGATTCTTGGGCTGTAAGTATTTAATTTCTGTTTCTTGAATCTAAAGCTGATTTGCATACAGTGT
TCGAATTGTTAACTTAGTTAGCGTTCTAGAGAATTGCGGCCCACAAGATGGTCTAGCTTGAATGTTTGAG
CTAGCTGTTGCTGCCAGTCTATAGGATGGCAACCTTAGCTACCTATACGCAAGTCTCTTGCTCCTGAGTC
ATCTAGATCGATTTACATGTTATGTGTATATACTTAGCGCTACATATACACCACACTCCTTTACATACAC
AAATTCATATTCTTACGGATCC

>SS1

AAATCTTCTAAACATTGTCGATTCCAGATCTTTTAAACCTGTCCCATACATTTGCTATATAATGAAATAC
CTCCCTATCCTCATCTCGGCTTTCGTAGCAGCCGTTGCTGCAGCTCCACAAGATCCGTCTGCTGTGGCAC
CTTTACTCCCTAGACAGTCTTGCGTACGTCTTGACTCATCGAATGACAGCCAGGAGATGAAACAACATGT
AGCTAATAATAGATAAAGGTATCATGTGTCAATTTCGGCAATGGGTTCTGTGGCGACAACTGTGGCAATT
CCTGGGCTGTAAGTATTTAATATCTGTTTCTCAAATCTAAAGCTAATGTGCATACAGTGTTCGGGCTGTT
AACTTAAGTAGCATTCTAGAGAATTATGGTCCACAAGACAGTCTAGCTTGAACACTATAGCTAGCTGTTG
CCACAAGTCTACAGGGTTGCAGTACTGATTGCCTATATAGGCAAACCTGTTGCTACCAAGTATTCTGGAC

TGTTTTTCTTGTTACTTATATACAATTGGCGCTATATATACACCACACTCATTTACATACCGCAAATCCA
AATCCTTGCAGATCC
>HHN10
AAATCTTCTAAACATTGTCGATTCCAGATCTTTTAAACCTGTCCCATACATTTGCTATATAATGAAATAC
CTCCCTATCCTCATCTCGGCTTTCGTAGCAGCCGTTGCTGCAGCTCCACAAGATCCGTCTGCTGTGGCAC
CTTTACTCCCTAGACAGTCTTGCGTACGTCTTGACTCATCGAATGACAGCCAGGAGATGAAACAACATGT
AGCTAATAATAGATAAAGGTATCATGTGTCAATTTCGGCAATGGGTTCTGTGGCGACAACTGTGGCAATT
CCTGGGCTGTAAGTATTTAATATCTGTTTCTCAAATCTAAAGCTAATGTGCATACAGTGTTCGGGCTGTT
AACTTAAGTAGCATTCTAGAGAATTATGGTCCACAAGACAGTCTAGCTTGAACACTATAGCTAGCTGTTG
CCACAAGTCTACAGGGTTGCAGTACTGATTGCCTATATAGGCAAACCTGTTGCTACCAAGTATTCTGGAC
TGTTTTTCTTGTTACTTATATACAATTGGCGCTATATATACACCACACTCATTTACATACCGCAAATCCA
AATCCTTGCAGATCC
>CSB6
AAATCTTCTAAACATTGTCGATTCCAGATCTTTTAAACCTGTCCCATACATTTGCTATATAATGAAATAC
CTCCCTATCCTCATCTCGGCTTTCGTAGCAGCCGTTGCTGCAGCTCCACAAGATCCGTCTGCTGTGGCAC
CTTTACTCCCTAGACAGTCTTGCGTACGTCTTGACTCATCGAATGACAGCCAGGAGATGAAACAACATGT
AGCTAATAATAGATAAAGGTATCATGTGTCAATTTCGGCAATGGGTTCTGTGGCGACAACTGTGGCAATT
CCTGGGCTGTAAGTATTTAATATCTGTTTCTCAAATCTAAAGCTAATGTGCATACAGTGTTCGGGCTGTT
AACTTAAGTAGCATTCTAGAGAATTATGGTCCACAAGACAGTCTAGCTTGAACACTATAGCTAGCTGTTG
CCACAAGTCTACAGGGTTGCAGTACTGATTGCCTATATAGGCAAACCTGTTGCTACCAAGTATTCTGGAC
TGTTTTTCTTGTTACTTATATACAATTGGCGCTATATATACACCACACTCATTTACATACCGCAAATCCA
AATCCTTGCAGATCC
>CSB5
AAATCTTCTAAACATTGTCGATTCCAGATCTTTTAAACCTGTCCCATACATTTGCTATATAATGAAATAC
CTCCCTATCCTCATCTCGGCTTTCGTAGCAGCCGTTGCTGCAGCTCCACAAGATCCGTCTGCTGTGGCAC
CTTTACTCCCTAGACAGTCTTGCGTACGTCTTGACTCATCGAATGACAGCCAGGAGATGAAACAACATGT
AGCTAATAATAGATAAAGGTATCATGTGTCAATTTCGGCAATGGGTTCTGTGGCGACAACTGTGGCAATT
CCTGGGCTGTAAGTATTTAATATCTGTTTCTCAAATCTAAAGCTAATGTGCATACAGTGTTCGGGCTGTT
AACTTAAGTAGCATTCTAGAGAATTATGGTCCACAAGACAGTCTAGCTTGAACACTATAGCTAGCTGTTG
CCACAAGTCTACAGGGTTGCAGTACTGATTGCCTATATAGGCAAACCTGTTGCTACCAAGTATTCTGGAC
TGTTTTTCTTGTTACTTATATACAATTGGCGCTATATATACACCACACTCATTTACATACCGCAAATCCA
AATCCTTGCAGATCC
>CSB3
AAATCTTCTAAACATTGTCGATTCCAGATCTTTTAAACCTGTCCCATACATTTGCTATATAATGAAATAC
CTCCCTATCCTCATCTCGGCTTTCGTAGCAGCCGTTGCTGCAGCTCCACAAGATCCGTCTGCTGTGGCAC
CTTTACTCCCTAGACAGTCTTGCGTACGTCTTGACTCATCGAATGACAGCCAGGAGATGAAACAACATGT
AGCTAATAATAGATAAAGGTATCATGTGTCAATTTCGGCAATGGGTTCTGTGGCGACAACTGTGGCAATT
CCTGGGCTGTAAGTATTTAATATCTGTTTCTCAAATCTAAAGCTAATGTGCATACAGTGTTCGGGCTGTT
AACTTAAGTAGCATTCTAGAGAATTATGGTCCACAAGACAGTCTAGCTTGAACACTATAGCTAGCTGTTG
CCACAAGTCTACAGGGTTGCAGTACTGATTGCCTATATAGGCAAACCTGTTGCTACCAAGTATTCTGGAC
TGTTTTTCTTGTTACTTATATACAATTGGCGCTATATATACACCACACTCATTTACATACCGCAAATCCA
AATCCTTGCAGATCC
>CSB16
AAATCTTCTAAACATTGTCGATTCCAGATCTTTTAAACCTGTCCCATACATTTGCTATATAATGAAATAC

CTCCCTATCCTCATCTCGGCTTTCGTAGCAGCCGTTGCTGCAGCTCCACAAGATCCGTCTGCTGTGGCAC
CTTTACTCCCTAGACAGTCTTGCGTACGTCTTGACTCATCGAATGACAGCCAGGAGATGAAACAACATGT
AGCTAATAATAGATAAAGGTATCATGTGTCAATTTCGGCAATGGGTTCTGTGGCGACAACTGTGGCAATT
CCTGGGCTGTAAGTATTTAATATCTGTTTCTCAAATCTAAAGCTAATGTGCATACAGTGTTCGGGCTGTT
AACTTAAGTAGCATTCTAGAGAATTATGGTCCACAAGACAGTCTAGCTTGAACACTATAGCTAGCTGTTG
CCACAAGTCTACAGGGTTGCAGTACTGATTGCCTATATAGGCAAACCTGTTGCTACCAAGTATTCTGGAC
TGTTTTTCTTGTTACTTATATACAATTGGCGCTATATATACACCACACTCATTTACATACCGCAAATCCA
AATCCTTGCAGATCC
>CSB11
AAATCTTCTAAACATTGTCGATTCCAGATCTTTTAAACCTGTCCCATACATTTGCTATATAATGAAATAC
CTCCCTATCCTCATCTCGGCTTTCGTAGCAGCCGTTGCTGCAGCTCCACAAGATCCGTCTGCTGTGGCAC
CTTTACTCCCTAGACAGTCTTGCGTACGTCTTGACTCATCGAATGACAGCCAGGAGATGAAACAACATGT
AGCTAATAATAGATAAAGGTATCATGTGTCAATTTCGGCAATGGGTTCTGTGGCGACAACTGTGGCAATT
CCTGGGCTGTAAGTATTTAATATCTGTTTCTCAAATCTAAAGCTAATGTGCATACAGTGTTCGGGCTGTT
AACTTAAGTAGCATTCTAGAGAATTATGGTCCACAAGACAGTCTAGCTTGAACACTATAGCTAGCTGTTG
CCACAAGTCTACAGGGTTGCAGTACTGATTGCCTATATAGGCAAACCTGTTGCTACCAAGTATTCTGGAC
TGTTTTTCTTGTTACTTATATACAATTGGCGCTATATATACACCACACTCATTTACATACCGCAAATCCA
AATCCTTGCAGATCC
>CSB05B
AAATCTTCTAAACATTGTCGATTCCAGATCTTTTAAACCTGTCCCATACATTTGCTATATAATGAAATAC
CTCCCTATCCTCATCTCGGCTTTCGTAGCAGCCGTTGCTGCAGCTCCACAAGATCCGTCTGCTGTGGCAC
CTTTACTCCCTAGACAGTCTTGCGTACGTCTTGACTCATCGAATGACAGCCAGGAGATGAAACAACATGT
AGCTAATAATAGATAAAGGTATCATGTGTCAATTTCGGCAATGGGTTCTGTGGCGACAACTGTGGCAATT
CCTGGGCTGTAAGTATTTAATATCTGTTTCTCAAATCTAAAGCTAATGTGCATACAGTGTTCGGGCTGTT
AACTTAAGTAGCATTCTAGAGAATTATGGTCCACAAGACAGTCTAGCTTGAACACTATAGCTAGCTGTTG
CCACAAGTCTACAGGGTTGCAGTACTGATTGCCTATATAGGCAAACCTGTTGCTACCAAGTATTCTGGAC
TGTTTTTCTTGTTACTTATATACAATTGGCGCTATATATACACCACACTCATTTACATACCGCAAATCCA
AATCCTTGCAGATCC
>CSB05A
AAATCTTCTAAACATTGTCGATTCCAGATCTTTTAAACCTGTCCCATACATTTGCTATATAATGAAATAC
CTCCCTATCCTCATCTCGGCTTTCGTAGCAGCCGTTGCTGCAGCTCCACAAGATCCGTCTGCTGTGGCAC
CTTTACTCCCTAGACAGTCTTGCGTACGTCTTGACTCATCGAATGACAGCCAGGAGATGAAACAACATGT
AGCTAATAATAGATAAAGGTATCATGTGTCAATTTCGGCAATGGGTTCTGTGGCGACAACTGTGGCAATT
CCTGGGCTGTAAGTATTTAATATCTGTTTCTCAAATCTAAAGCTAATGTGCATACAGTGTTCGGGCTGTT
AACTTAAGTAGCATTCTAGAGAATTATGGTCCACAAGACAGTCTAGCTTGAACACTATAGCTAGCTGTTG
CCACAAGTCTACAGGGTTGCAGTACTGATTGCCTATATAGGCAAACCTGTTGCTACCAAGTATTCTGGAC
TGTTTTTCTTGTTACTTATATACAATTGGCGCTATATATACACCACACTCATTTACATACCGCAAATCCA
AATCCTTGCAGATCC
>CKT1
AAATCTTCTAAACATTGTCGATTCCAGATCTTTTAAACCTGTCCCATACATTTGCTATATAATGAAATAC
CTCCCTATCCTCATCTCGGCTTTCGTAGCAGCCGTTGCTGCAGCTCCACAAGATCCGTCTGCTGTGGCAC
CTTTACTCCCTAGACAGTCTTGCGTACGTCTTGACTCATCGAATGACAGCCAGGAGATGAAACAACATGT
AGCTAATAATAGATAAAGGTATCATGTGTCAATTTCGGCAATGGGTTCTGTGGCGACAACTGTGGCAATT
CCTGGGCTGTAAGTATTTAATATCTGTTTCTCAAATCTAAAGCTAATGTGCATACAGTGTTCGGGCTGTT

AACTTAAGTAGCATTCTAGAGAATTATGGTCCACAAGACAGTCTAGCTTGAACACTATAGCTAGCTGTTG
CCACAAGTCTACAGGGTTGCAGTACTGATTGCCTATATAGGCAAACCTGTTGCTACCAAGTATTCTGGAC
TGTTTTTCTTGTTACTTATATACAATTGGCGCTATATATACACCACACTCATTTACATACCGCAAATCCA
AATCCTTGCAGATCC
>CKT05F
AAATCTTCTAAACATTGTCGATTCCAGATCTTTTAAACCTGTCCCATACATTTGCTATATAATGAAATAC
CTCCCTATCCTCATCTCGGCTTTCGTAGCAGCCGTTGCTGCAGCTCCACAAGATCCGTCTGCTGTGGCAC
CTTTACTCCCTAGACAGTCTTGCGTACGTCTTGACTCATCGAATGACAGCCAGGAGATGAAACAACATGT
AGCTAATAATAGATAAAGGTATCATGTGTCAATTTCGGCAATGGGTTCTGTGGCGACAACTGTGGCAATT
CCTGGGCTGTAAGTATTTAATATCTGTTTCTCAAATCTAAAGCTAATGTGCATACAGTGTTCGGGCTGTT
AACTTAAGTAGCATTCTAGAGAATTATGGTCCACAAGACAGTCTAGCTTGAACACTATAGCTAGCTGTTG
CCACAAGTCTACAGGGTTGCAGTACTGATTGCCTATATAGGCAAACCTGTTGCTACCAAGTATTCTGGAC
TGTTTTTCTTGTTACTTATATACAATTGGCGCTATATATACACCACACTCATTTACATACCGCAAATCCA
AATCCTTGCAGATCC
>CKT05C
AAATCTTCTAAACATTGTCGATTCCAGATCTTTTAAACCTGTCCCATACATTTGCTATATAATGAAATAC
CTCCCTATCCTCATCTCGGCTTTCGTAGCAGCCGTTGCTGCAGCTCCACAAGATCCGTCTGCTGTGGCAC
CTTTACTCCCTAGACAGTCTTGCGTACGTCTTGACTCATCGAATGACAGCCAGGAGATGAAACAACATGT
AGCTAATAATAGATAAAGGTATCATGTGTCAATTTCGGCAATGGGTTCTGTGGCGACAACTGTGGCAATT
CCTGGGCTGTAAGTATTTAATATCTGTTTCTCAAATCTAAAGCTAATGTGCATACAGTGTTCGGGCTGTT
AACTTAAGTAGCATTCTAGAGAATTATGGTCCACAAGACAGTCTAGCTTGAACACTATAGCTAGCTGTTG
CCACAAGTCTACAGGGTTGCAGTACTGATTGCCTATATAGGCAAACCTGTTGCTACCAAGTATTCTGGAC
TGTTTTTCTTGTTACTTATATACAATTGGCGCTATATATACACCACACTCATTTACATACCGCAAATCCA
AATCCTTGCAGATCC
>C68-8
AAATCTTCTAAACATTGTCGATTCCAGATCTTTTAAACCTGTCCCATACATTTGCTATATAATGAAATAC
CTCCCTATCCTCATCTCGGCTTTCGTAGCAGCCGTTGCTGCAGCTCCACAAGATCCGTCTGCTGTGGCAC
CTTTACTCCCTAGACAGTCTTGCGTACGTCTTGACTCATCGAATGACAGCCAGGAGATGAAACAACATGT
AGCTAATAATAGATAAAGGTATCATGTGTCAATTTCGGCAATGGGTTCTGTGGCGACAACTGTGGCAATT
CCTGGGCTGTAAGTATTTAATATCTGTTTCTCAAATCTAAAGCTAATGTGCATACAGTGTTCGGGCTGTT
AACTTAAGTAGCATTCTAGAGAATTATGGTCCACAAGACAGTCTAGCTTGAACACTATAGCTAGCTGTTG
CCACAAGTCTACAGGGTTGCAGTACTGATTGCCTATATAGGCAAACCTGTTGCTACCAAGTATTCTGGAC
TGTTTTTCTTGTTACTTATATACAATTGGCGCTATATATACACCACACTCATTTACATACCGCAAATCCA
AATCCTTGCAGATCC
>C68-6
AAATCTTCTAAACATTGTCGATTCCAGATCTTTTAAACCTGTCCCATACATTTGCTATATAATGAAATAC
CTCCCTATCCTCATCTCGGCTTTCGTAGCAGCCGTTGCTGCAGCTCCACAAGATCCGTCTGCTGTGGCAC
CTTTACTCCCTAGACAGTCTTGCGTACGTCTTGACTCATCGAATGACAGCCAGGAGATGAAACAACATGT
AGCTAATAATAGATAAAGGTATCATGTGTCAATTTCGGCAATGGGTTCTGTGGCGACAACTGTGGCAATT
CCTGGGCTGTAAGTATTTAATATCTGTTTCTCAAATCTAAAGCTAATGTGCATACAGTGTTCGGGCTGTT
AACTTAAGTAGCATTCTAGAGAATTATGGTCCACAAGACAGTCTAGCTTGAACACTATAGCTAGCTGTTG
CCACAAGTCTACAGGGTTGCAGTACTGATTGCCTATATAGGCAAACCTGTTGCTACCAAGTATTCTGGAC
TGTTTTTCTTGTTACTTATATACAATTGGCGCTATATATACACCACACTCATTTACATACCGCAAATCCA
AATCCTTGCAGATCC

>CSR12
AAATCTTCTAAACATTGTCGATTCCAGATCTTTTAAACCTGTCCCATACATTTGCTATATAATGAAATAC
CTCCCTATCCTCATCTCGGCTTTCGTAGCAGCCGTTGCTGCAGCTCCACAAGATCCGTCTGCTGTGGCAC
CTTTACTCCCTAGACAGTCTTGCGTACGTCTTGACTCATCGAATGACAGCCAGGAGATGAAACAACATGT
AGCTAATAATAGATAAAGGTATCATGTGTCAATTTCGGCAATGGGTTCTGTGGCGACAACTGTGGCAATT
CCTGGGCTGTAAGTATTTAATATCTGTTTCTCAAATCTAAAGCTAATGTGCATACAGTGTTCGGGCTGTT
AACTTAAGTAGCATTCTAGAGAATTATGGTCCACAAGACAGTCTAGCTTGAACACTATAGCTAGCTGTTG
CCACAAGTCTACAGGGTTGCAGTACTGATTGCCTATATAGGCAAACCTGTTGCTACCAAGTATTCTGGAC
TGTTTTTCTTGTTACTTATATACAATTGGCGCTATATATACACCACACTCATTTACATACCGCAAATCCA
AATCCTTGCAGATCC
>CSB16R1
AAATCTTCTAAACATTGTCGATTCCAGATCTTTTAAACCTGTCCCATACATTTGCTATATAATGAAATAC
CTCCCTATCCTCATCTCGGCTTTCGTAGCAGCCGTTGCTGCAGCTCCACAAGATCCGTCTGCTGTGGCAC
CTTTACTCCCTAGACAGTCTTGCGTACGTCTTGACTCATCGAATGACAGCCAGGAGATGAAACAACATGT
AGCTAATAATAGATAAAGGTATCATGTGTCAATTTCGGCAATGGGTTCTGTGGCGACAACTGTGGCAATT
CCTGGGCTGTAAGTATTTAATATCTGTTTCTCAAATCTAAAGCTAATGTGCATACAGTGTTCGGGCTGTT
AACTTAAGTAGCATTCTAGAGAATTATGGTCCACAAGACAGTCTAGCTTGAACACTATAGCTAGCTGTTG
CCACAAGTCTACAGGGTTGCAGTACTGATTGCCTATATAGGCAAACCTGTTGCTACCAAGTATTCTGGAC
TGTTTTTCTTGTTACTTATATACAATTGGCGCTATATATACACCACACTCATTTACATACCGCAAATCCA
AATCCTTGCAGATCC
>CSB4
AAATCTTCTAAACATTGTCGATTCCAGATCTTTTAAACCTGTCCCATACATTTGCTATATAATGAAATAC
CTCCCTATCCTCATCTCGGCTTTCGTAGCAGCCGTTGCTGCAGCTCCACAAGATCCGTCTGCTGTGGCAC
CTTTACTCCCTAGACAGTCTTGCGTACGTCTTGACTCATCGAATGACAGCCAGGAGATGAAACAACATGT
AGCTAATAATAGATAAAGGTATCATGTGTCAATTTCGGCAATGGGTTCTGTGGCGACAACTGTGGCAATT
CCTGGGCTGTAAGTATTTAATATCTGTTTCTCAAATCTAAAGCTAATGTGCATACAGTGTTCGGGCTGTT
AACTTAAGTAGCATTCTAGAGAATTATGGTCCACAAGACAGTCTAGCTTGAACACTATAGCTAGCTGTTG
CCACAAGTCTACAGGGTTGCAGTACTGATTGCCTATATAGGCAAACCTGTTGCTACCAAGTATTCTGGAC
TGTTTTTCTTGTTACTTATATACAATTGGCGCTATATATACACCACACTCATTTACATACCGCAAATCCA
AATCCTTGCAGATCC
>CSB1
AAATCTTCTAAACATTGTCGATTCCAGATCTTTTAAACCTGTCCCATACATTTGCTATATAATGAAATAC
CTCCCTATCCTCATCTCGGCTTTCGTAGCAGCCGTTGCTGCAGCTCCACAAGATCCGTCTGCTGTGGCAC
CTTTACTCCCTAGACAGTCTTGCGTACGTCTTGACTCATCGAATGACAGCCAGGAGATGAAACAACATGT
AGCTAATAATAGATAAAGGTATCATGTGTCAATTTCGGCAATGGGTTCTGTGGCGACAACTGTGGCAATT
CCTGGGCTGTAAGTATTTAATATCTGTTTCTCAAATCTAAAGCTAATGTGCATACAGTGTTCGGGCTGTT
AACTTAAGTAGCATTCTAGAGAATTATGGTCCACAAGACAGTCTAGCTTGAACACTATAGCTAGCTGTTG
CCACAAGTCTACAGGGTTGCAGTACTGATTGCCTATATAGGCAAACCTGTTGCTACCAAGTATTCTGGAC
TGTTTTTCTTGTTACTTATATACAATTGGCGCTATATATACACCACACTCATTTACATACCGCAAATCCA
AATCCTTGCAGATCC
>LP138
TATCGTTCGATCTTTGAGACCTGTCCTATATATTTGCTACACAATGAAATACCTCCCTATCTTCATCTCT
GCTTTTGTAGCAGCCGTTGCTGCAGTTCCGCAGGGTCCGTCTGCTGCGGCAGCAGCTATTCTTCCTAGAC
AGTCTTGCGTACGTCTTGACTTATCAAAGCACGATCAGAAAAAATGAAACAATTTGTAACTAATAATTGG

TAAAGGTATCATGTGTCGATTTCGGCAACGGGTTCTGTGGCGACAACTGTGGTAATTCCTGGGCTGTAAG
TATTTAATATCTGTTTCTTGATTCTAAAGCTAATTTGCATACAGTGTTCGGGTTGTTAGCTTAGGTAGCG
TTCTAGATAATTGTGGCCTACAAGATAGTGTAGCTTGAATATTTGAGCTGGCTGCCGCTACAAGTCTAAA
GGATGGCAACCTTAGCTACCTATACGCAAGTATCTTACTTCTAGGTCGTTTAGACCAATTTGCATATTAT
GTGTATATACTTAGCGCTACATATACACCACACTCCTTTACAAACACAAATTTATATTCTTACGGACCCT
AAAATTTGCTCTACAAAACGTGAGATAGCGTGAAAAGGCGGTTTGGCCTATAGTTCTCTTTTAACTAGTT
CTGTATCTTGCTTTCTGTATCCCAAGCATATTTACGCAAAGTTTAAAAGAGCCACCCGGTTCTGAAATCC
GTTTGTTGGAT
>CC01
TACGTTTCTTAAGCACTATCAGTTCGAAATCTTTGAGACCTGTCCTATATATTTGCTACACAATGAAATA
CCTCCCTATCTTCATCTCTGCTTTTGTAGCAGCCGTTGCTGCAGTTCCGCAGGGTCCGTCTGCTGCGGCA
GCAGCTATTCTTCCTAGACAGTCTTGCGTACGTCTTGACTTATCAAAGCACGATCAGAAAAAATGAAACA
ATTTGTAACTAATAATTGGTAAAGGTATCACGTGTCGATTTCGGCAACGGGTTCTGTGGCGACAACTGTG
GTAATTCCTGGGCTGTAAGTATTTAATATCTGTTTCTTGATTCTAAAGCTAATTTGCATACAGTGTTCGG
GTTGTTAGCTTAGGTAGCGTTCTAGATAATTGTGGCCTACAAGATAGTGTAGCTTGAATATTTGAGCTGG
CTGCCGCTACAAGTCTAAAGGATGGCAACCTTAGCTACCTATACGCAAGTATCTTACTTCTAGGTCGCTT
AGACCAATTTGCATATTATGTGTATATACTTAGCGCTACATATACACCACACTCCTTTACATACACAAAT
TTATATTCTTACGGATCC
>ATI13
TACGTTTCTTAAGCACTATCAGTTCGAAATCTTTGAGACCTGTCCTATATATTTGCTACACAATGAAATA
CCTCCCTATCTTCATCTCTGCTTTTGTAGCAGCCGTTGCTGCAGTTCCGCAGGGTCCGTCTGCTGCGGCA
GCAGCTATTCTTCCTAGACAGTCTTGCGTACGTCTTGACTTATCAAAGCACGATCAGAAAAAATGAAACA
ATTTGTAACTAATAATTGGTAAAGGTATCATGTGTCGATTTCGGCAACGGGTTCTGTGGCGACAACTGTG
GTAATTCCTGGGCTGTAAGTATTTAATATCTGTTTCTTGATTCTAAAGCTAATTTGCATACAGTGTTCGG
GTTGTTAGCTTAGGTAGCGTTCTAGATAATTGTGGCCTACAAGATAGTGTAGCTTGAATATTTGAGCTGG
CTGCCGCTACAAGTCTAAAGGATGGCAACCTTAGCTACCTATACGCAAGTATCTTACTTCTAGGTCGTTT
AGACCAATTTGCATATTATGTGTATATACTTAGCGCTACATATACACCACACTCCTTTACAAACACAAAT
TTATATTCTTACGGATCC
>JMP2020
TACGTTTCTTAAGCACTATCAGTTCGAAATCTTTGAGACCTGTCCTATATATTTGCTACACAATGAAATA
CCTCCCTATCTTCATCTCTGCTTTTGTAGCAGCCGTTGCTGCAGTTCCGCAGGGTCCGTCTGCTGCGGCA
GCAGCTATTCTTCCTAGACAGTCTTGCGTACGTCTTGACTTATCAAAGCACGATCAGAAAAAATGAAACA
ATTTGTAACTAATAATTGGTAAAGGTATCATGTGTCGATTTCGGCAACGGGTTCTGTGGCGACAACTGTG
GTAATTCCTGGGCTGTAAGTATTTAATATCTGTTTCTTGATTCTAAAGCTAATTTGCATACAGTGTTCGG
GTTGTTAGCTTAGGTAGCGTTCTAGATAATTGTGGCCTACAAGATAGTGTAGCTTGAATATTTGAGCTGG
CTGCCGCTACAAGTCTAAAGGATGGCAACCTTAGCTACCTATACGCAAGTATCTTACTTCTAGGTCGTTT
AGACCAATTTGCATATTATGTGTATGTACTTAGCGCTACATATACACCACACTCCTTTACATACACAAAT
TTATATTCTTACGGATCC
>GSO2
TACGTTTCTTAAGCACTATCAGTTCGAAATCTTTGAGACCTGTCCTATATATTTGCTACACAATGAAATA
CCTCCCTATCTTCATCTCTGCTTTTGTAGCAGCCGTTGCTGCAGTTCCGCAGGGTCCGTCTGCTGCGGCA
GCAGCTATTCTTCCTAGACAGTCTTGCGTACGTCTTGACTTGTCAAAGCACGATCAGAAAAAATGAAACA
AATTGTAACTAATAATTGGTAAAGGTATCATGTGTCGATTTCGGCAACGGGTTCTGTGGCGACAACTGTG
GTAATTCCTGGGCTGTAAGTATTTAATATCTGTTTCTTGATTCTAAAGCTAATTTGCATACAGTGTTCGG

GTTGTTAGCTTAGGTAGCGTTCTAGATAATTGTGGCCTACAAGATAGTGTAGCTTGAATATTTGAGCTGG
CTGCCGCTACAAGTCTAAAGGATGGCAACCTTAGCTACCTATACGCAAGTATCTTACTTCTAGGTCGTTT
AGACCAATTTGCATATTATGTGTATGTACTTAGCGCTACATATACACCACACTCCTTTACATACACAAAT
TTATATTCTTACGGATCC
>PB
TACGTTTCTTAAGCACTATCAGTTCGAAATCTTTGAGACCTGTCCTATATATTTGCTACACAATGAAATA
CCTCCCTATCTTCATCTCTGCTTTTGTAGCAGCCGTTGCTGCAGTTCCGCAGGGTCCGTCTGCTGCGGCA
GCAGCTATTCTTCCTAGACAGTCTTGCGTACGTCTTGACTTATCAAAGCACGATCAGAAAAAATGAAACA
ATTTGTAACTAATAATTGGTAAAGGTATCATGTGTCGATTTCGGCAACGGGTTCTGTGGCGACAACTGTG
GTAATTCCTGGGCTGTAAGTATTTAATATCTGTTTCTTGATTCTAAAGCTAATTTGCATACAGTGTTCGG
GTTGTTAGCTTAGGTAGCGTTCTAGATAATTGTGGCCTACAAGATAGTGTAGCTTGAATATTTGAGCTGG
CTGCCGCTACAAGTCTAAAGGATGGCAACCTTAGCTACCTATACGCAAGTATCTTACTTCTAGGTCGTTT
AGACCAATTTGCATATTATGTGTATATACTTAGCGCTACATATACACCACACTCCTTTACATACACAAAT
TTATATTCTTACGGATCC
>RUD
TACGTTTCTTAAGCACTATCAGTTCGAAATCTTTGAGACCTGTCCTATATATTTGCTACACAATGAAATA
CCTCCCTATCTTCATCTCTGCTTTTGTAGCAGCCGTTGCTGCAGTTCCGCAGGGTCCGTCTGCTGCGGCA
GCAGCTATTCTTCCTAGACAGTCTTGCGTACGTCTTGACTTATCAAAGCACGATCAGAAAAAATGAAACA
ATTTGTAACTAATAATTGGTAAAGGTATCATGTGTCGATTTCGGCAACGGGTTCTGTGGCGACAACTGTG
GTAATTCCTGGGCTGTAAGTATTTAATATCTGTTTCTTGATTCTAAAGCTAATTTGCATACAGTGTTCGG
GTTGTTAGCTTAGGTAGCGTTCTAGATAATTGTGGCCTACAAGATAGTGTAGCTTGAATATTTGAGCTGG
CTGCCGCTACAAGTCTAAAGGATGGCAACCTTAGCTACCTATACGCAAGTATCTTACTTCTAGGTCGTTT
AGACCAATTTGCATATTATGTGTATATACTTAGCGCTACATATACACCACACTCCTTTACATACACAAAT
TTATATTCTTACGGATCC
>IA
TACGTTTCTTAAGCACTATCAGTTCGAAATCTTTGAGACCTGTCCTATATATTTGCTACACAATGAAATA
CCTCCCTATCTTCATCTCTGCTTTTGTAGCAGCCGTTGCTGCAGTTCCGCAGGGTCCGTCTGCTGCGGCA
GCAGCTATTCTTCCTAGACAGTCTTGCGTACGTCTTGACTTATCAAAGCACGATCAGAAAAAATGAAACA
ATTTGTAACTAATAATTGGTAAAGGTATCATGTGTCGATTTCGGCAACGGGTTCTGTGGCGACAACTGTG
GTAATTCCTGGGCTGTAAGTATTTAATATCTGTTTCTTGATTCTAAAGCTAATTTGCATACAGTGTTCGG
GTTGTTAGCTTAGGTAGCGTTCTAGATAATTGTGGCCTACAAGATAGTGTAGCTTGAATATTTGAGCTGG
CTGCCGCTACAAGTCTAAAGGATGGCAACCTTAGCTACCTATACGCAAGTATCTTACTTCTAGGTCGTTT
AGACCAATTTGCATATTATGTGTATATACTTAGCGCTACATATACACCACACTCCTTTACATACACAAAT
TTATATTCTTACGGATCC
>EDIG
TACGTTTCTTAAGCACTATCAGTTCGAAATCTTTGAGACCTGTCCTATATATTTGCTACACAATGAAATA
CCTCCCTATCTTCATCTCTGCTTTTGTAGCAGCCGTTGCTGCAGTTCCGCAGGGTCCGTCTGCTGCGGCA
GCAGCTATTCTTCCTAGACAGTCTTGCGTACGTCTTGACTTATCAAAGCACGATCAGAAAAAATGAAACA
ATTTGTAACTAATAATTGGTAAAGGTATCATGTGTCGATTTCGGCAACGGGTTCTGTGGCGACAACTGTG
GTAATTCCTGGGCTGTAAGTATTTAATATCTGTTTCTTGATTCTAAAGCTAATTTGCATACAGTGTTCGG
GTTGTTAGCTTAGGTAGCGTTCTAGATAATTGTGGCCTACAAGATAGTGTAGCTTGAATATTTGAGCTGG
CTGCCGCTACAAGTCTAAAGGATGGCAACCTTAGCTACCTATACGCAAGTATCTTACTTCTAGGTCGTTT
AGACCAATTTGCATATTATGTGTATATACTTAGCGCTACATATACACCACACTCCTTTACATACACAAAT
TTATATTCTTACGGATCC

>CMENY
TACGTTTCTTAAGCACTATCAGTTCGAAATCTTTGAGACCTGTCCTATATATTTGCTACACAATGAAATA
CCTCCCTATCTTCATCTCTGCTTTTGTAGCAGCCGTTGCTGCAGTTCCGCAGGGTCCGTCTGCTGCGGCA
GCAGCTATTCTTCCTAGACAGTCTTGCGTACGTCTTGACTTATCAAAGCACGATCAGAAAAAATGAAACA
ATTTGTAACTAATAATTGGTAAAGGTATCATGTGTCGATTTCGGCAACGGGTTCTGTGGCGACAACTGTG
GTAATTCCTGGGCTGTAAGTATTTAATATCTGTTTCTTGATTCTAAAGCTAATTTGCATACAGTGTTCGG
GTTGTTAGCTTAGGTAGCGTTCTAGATAATTGTGGCCTACAAGATAGTGTAGCTTGAATATTTGAGCTGG
CTGCCGCTACAAGTCTAAAGGATGGCAACCTTAGCTACCTATACGCAAGTATCTTACTTCTAGGTCGTTT
AGACCAATTTGCATATTATGTGTATATACTTAGCGCTACATATACACCACACTCCTTTACATACACAAAT
TTATATTCTTACGGATCC
>ATI17
TACGTTTCTTAAGCACTATCAGTTCGAAATCTTTGAGACCTGTCCTATATATTTGCTACACAATGAAATA
CCTCCCTATCTTCATCTCTGCTTTTGTAGCAGCCGTTGCTGCAGTTCCGCAGGGTCCGTCTGCTGCGGCA
GCAGCTATTCTTCCTAGACAGTCTTGCGTACGTCTTGACTTATCAAAGCACGATCAGAAAAAATGAAACA
ATTTGTAACTAATAATTGGTAAAGGTATCATGTGTCGATTTCGGCAACGGGTTCTGTGGCGACAACTGTG
GTAATTCCTGGGCTGTAAGTATTTAATATCTGTTTCTTGATTCTAAAGCTAATTTGCATACAGTGTTCGG
GTTGTTAGCTTAGGTAGCGTTCTAGATAATTGTGGCCTACAAGATAGTGTAGCTTGAATATTTGAGCTGG
CTGCCGCTACAAGTCTAAAGGATGGCAACCTTAGCTACCTATACGCAAGTATCTTACTTCTAGGTCGTTT
AGACCAATTTGCATATTATGTGTATATACTTAGCGCTACATATACACCACACTCCTTTACATACACAAAT
TTATATTCTTACGGATCC
>CBS
TACGTTTCTTAAGCACTATCAGTTCGAAATCTTTGAGACCTGTCCTATATATTTGCTACACAATGAAATA
CCTCCCTATCTTCATCTCTGCTTTTGTAGCAGCCGTTGCTGCAGTTCCGCAGGGTCCGTCTGCTGCGGCA
GCAGCTATTCTTCCTAGACAGTCTTGCGTACGTCTTGACTTATCAAAGCACGATCAGAAAAAATGAAACA
ATTTGTAACTAATAATTGGTAAAGGTATCATGTGTCGATTTCGGCAACGGGTTCTGTGGCGACAACTGTG
GTAATTCCTGGGCTGTAAGTATTTAATATCTGTTTCTTGATTCTAAAGCTAATTTGCATACAGTGTTCGG
GTTGTTAGCTTAGGTAGCGTTCTAGATAATTGTGGCCTACAAGATAGTGTAGCTTGAATATTTGAGCTGG
CTGCCGCTACAAGTCTAAAGGATGGCAACCTTAGCTACCTATACGCAAGTATCTTACTTCTAGGTCGTTT
AGACCAATTTGCATATTATGTGTATATACTTAGCGCTACATATACACCACACTCCTTTACATACACAAAT
TTATATTCTTACGGATCC
>ATI11
TACGTTTCTTAAGCACTATCAGTTCGAAATCTTTGAGACCTGTCCTATATATTTGCTACACAATGAAATA
CCTCCCTATCTTCATCTCTGCTTTTGTAGCAGCCGTTGCTGCAGTTCCGCAGGGTCCGTCTGCTGCGGCA
GCAGCTATTCTTCCTAGACAGTCTTGCGTACGTCTTGACTTATCAAAGCACGATCAGAAAAAATGAAACA
ATTTGTAACTAATAATTGGTAAAGGTATCATGTGTCGATTTCGGCAACGGGTTCTGTGGCGACAACTGTG
GTAATTCCTGGGCTGTAAGTATTTAATATCTGTTTCTTGATTCTAAAGCTAATTTGCATACAGTGTTCGG
GTTGTTAGCTTAGGTAGCGTTCTAGATAATTGTGGCCTACAAGATAGTGTAGCTTGAATATTTGAGCTGG
CTGCCGCTACAAGTCTAAAGGATGGCAACCTTAGCTACCTATACGCAAGTATCTTACTTCTAGGTCGTTT
AGACCAATTTGCATATTATGTGTATATACTTAGCGCTACATATACACCACACTCCTTTACATACACAAAT
TTATATTCTTACGGATCC
>777AA
TACGTTTCTTAAGCACTATCAGTTCGAAATCTTTGAGACCTGTCCTATATATTTGCTACACAATGAAATA
CCTCCCTATCTTCATCTCTGCTTTTGTAGCAGCCGTTGCTGCAGTTCCGCAGGGTCCGTCTGCTGCGGCA
GCAGCTATTCTTCCTAGACAGTCTTGCGTACGTCTTGACTTATCAAAGCACGATCAGAAAAAATGAAACA

ATTTGTAACTAATAATTGGTAAAGGTATCATGTGTCGATTTCGGCAACGGGTTCTGTGGCGACAACTGTG
GTAATTCCTGGGCTGTAAGTATTTAATATCTGTTTCTTGATTCTAAAGCTAATTTGCATACAGTGTTCGG
GTTGTTAGCTTAGGTAGCGTTCTAGATAATTGTGGCCTACAAGATAGTGTAGCTTGAATATTTGAGCTGG
CTGCCGCTACAAGTCTAAAGGATGGCAACCTTAGCTACCTATACGCAAGTATCTTACTTCTAGGTCGTTT
AGACCAATTTGCATATTATGTGTATATACTTAGCGCTACATATACACCACACTCCTTTACATACACAAAT
TTATATTCTTACGGATCC
>493AA
TACGTTTCTTAAGCACTATCAGTTCGAAATCTTTGAGACCTGTCCTATATATTTGCTACACAATGAAATA
CCTCCCTATCTTCATCTCTGCTTTTGTAGCAGCCGTTGCTGCAGTTCCGCAGGGTCCGTCTGCTGCGGCA
GCAGCTATTCTTCCTAGACAGTCTTGCGTACGTCTTGACTTATCAAAGCACGATCAGAAAAAATGAAACA
ATTTGTAACTAATAATTGGTAAAGGTATCATGTGTCGATTTCGGCAACGGGTTCTGTGGCGACAACTGTG
GTAATTCCTGGGCTGTAAGTATTTAATATCTGTTTCTTGATTCTAAAGCTAATTTGCATACAGTGTTCGG
GTTGTTAGCTTAGGTAGCGTTCTAGATAATTGTGGCCTACAAGATAGTGTAGCTTGAATATTTGAGCTGG
CTGCCGCTACAAGTCTAAAGGATGGCAACCTTAGCTACCTATACGCAAGTATCTTACTTCTAGGTCGTTT
AGACCAATTTGCATATTATGTGTATATACTTAGCGCTACATATACACCACACTCCTTTACATACACAAAT
TTATATTCTTACGGATCC
>YN49
TCTTCTAAACATTGTCGATTCCAGATCTTTTAAACCTGTCCCATACATTTGCTATATAAT
GAAATACCTCCCTATCCTCATCTCGGCTTTCGTAGCAGCCGTTGCTGCAGCTCCACAAGA
TCCGTCTGCTGTGGCACCTTTACTCCCTAGACAGTCTTGCGTACGTCTTGACTCATCGAA
TGACAGCCAGGAGATGAAACAACATGTAGCTAATAATAGATAAAGGTATCATGTGTCAAT
TTCGGCAATGGGTTCTGTGGCGACAACTGTGGCAATTCCTGGGCTGTAAGTATTTAATAT
CTGTTTCTCAAATCTAAAGCTAATGTGCATACAGTGTTCGGGCTGTTAACTTAAGTAGCA
TTCTAGAGAATTATGGTCCACAAGACAGTCTAGCTTGAACACTATAGCTAGCTGTTGCCA
CAAGTCTACAGGGTTGCAGTACTGATTGCCTATATAGGCAAACCTGTTGCTACCAAGTAT
TCTGGACTGTTTTTCTTGTTACTTATATACAATTGGCGC-TATATA-TACACCACACTCA
TTTACATACCGCAAATCCAAATCCTTGCAGATCCTAAACTTTGCTCTGCATAACGTGAGA
TCACACAAGCAGGGAACTTGGCCAGTAGTTC
