# Supplementary figures and images for: Genomic Characteristics and Comparative Genomics Analysis of Two Chinese Corynespora cassiicola Strains Causing Corynespora Leaf Fall (CLF) Disease
Source: J Fungi (Basel). 2021 Jun 16;7(6):485. doi: 10.3390/jof7060485 (PMC8235470; doi:10.3390/jof7060485)

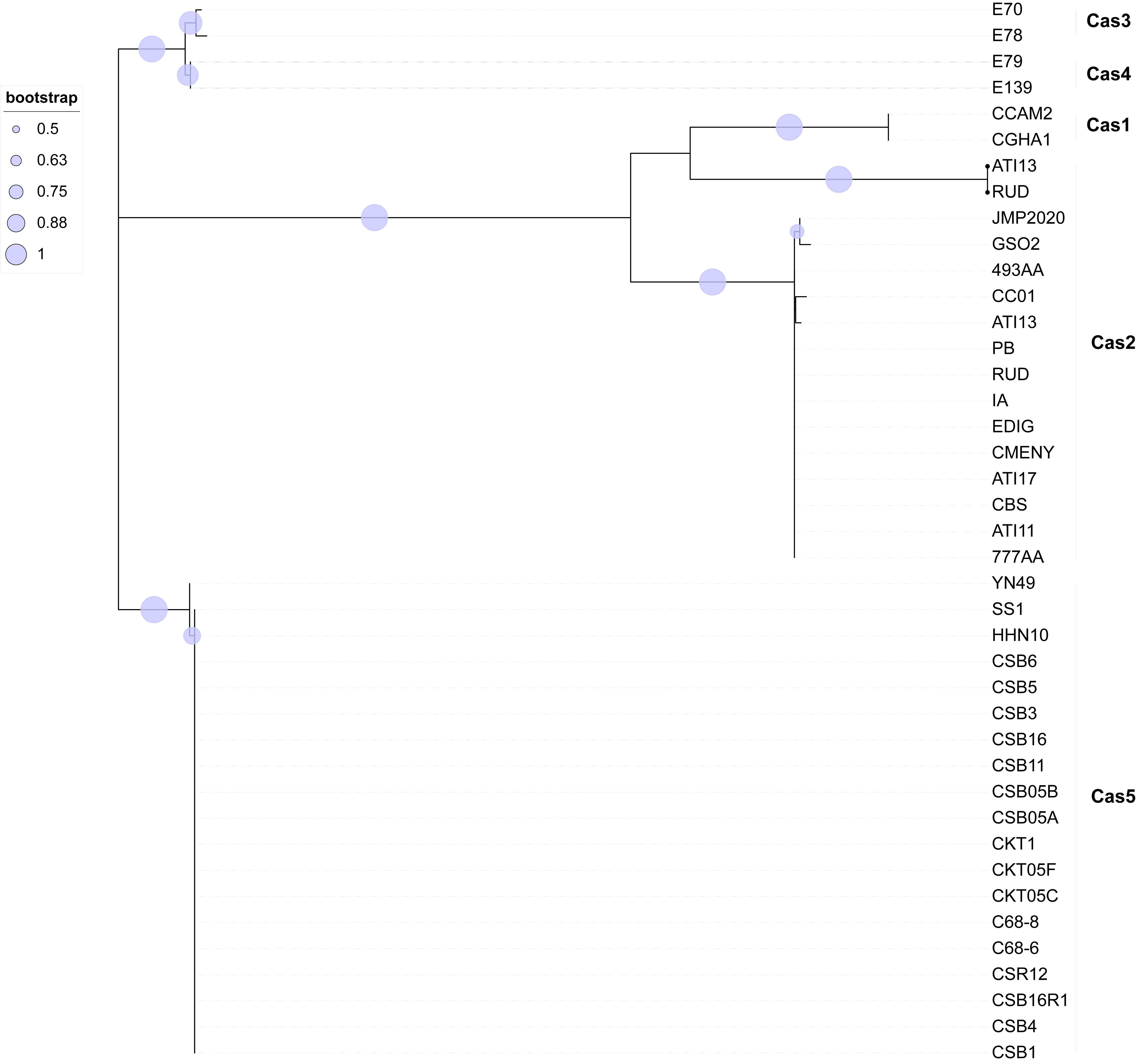

Supplement: Supplementary file 1 [file jof-07-00485-s001.zip › jof-1246405-supplementary/Figure S1.jpg]

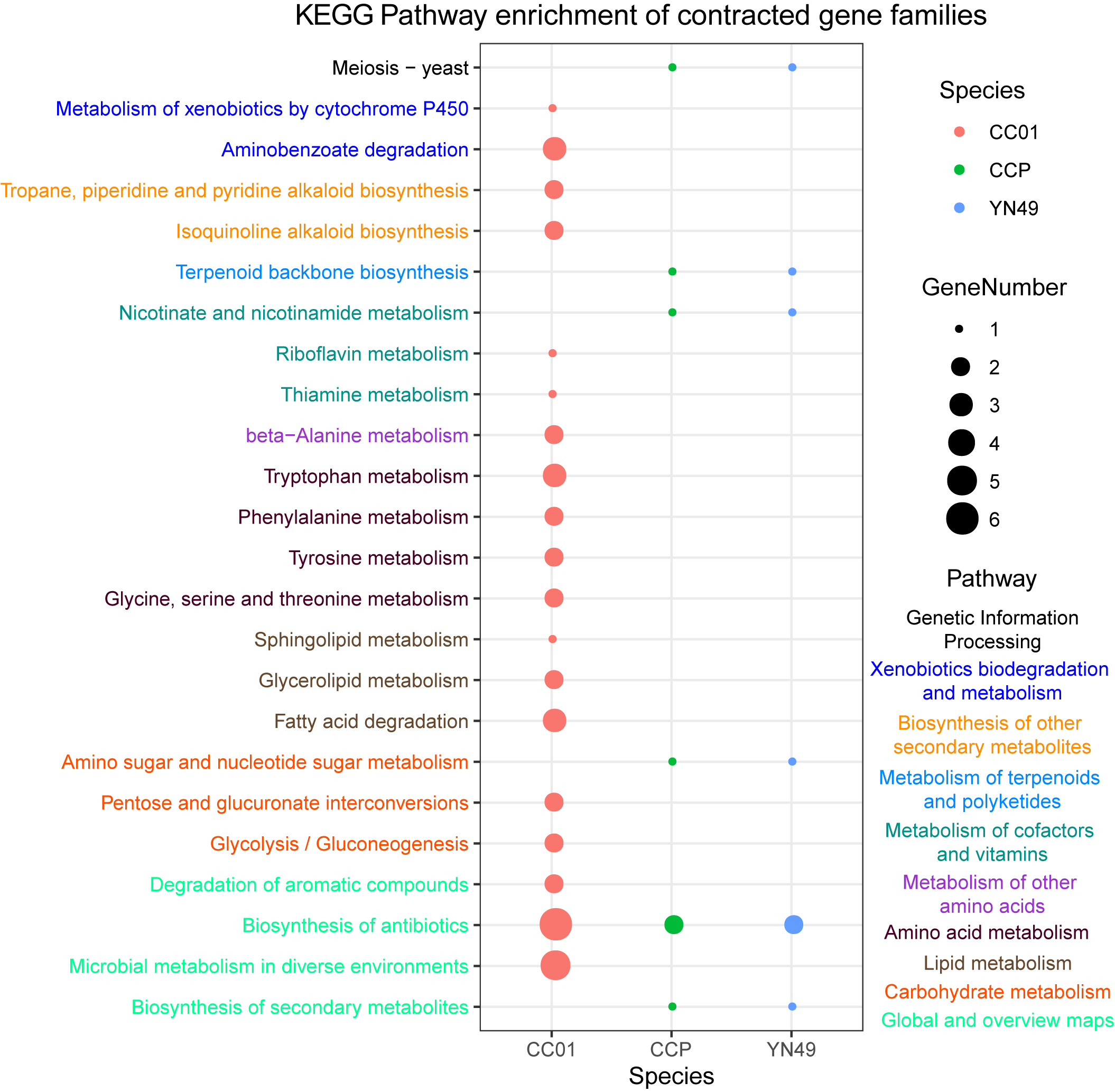

Supplement: Supplementary file 1 [file jof-07-00485-s001.zip › jof-1246405-supplementary/Figure S2.jpg]
